# Mesenchymal stem cell derived exosomes and injectable platelet-rich fibrin enhance SDFT healing in a donkey tendonitis model

**DOI:** 10.1038/s41598-026-44967-7

**Published:** 2026-04-10

**Authors:** Mahmoud Najeb, Alaa Samy, Awad Rizk, Esam Mosbah, Iman Ibrahim, Gamal Karrouf

**Affiliations:** 1 Department of Surgery, Anesthesiology, and Radiology, Faculty of Veterinary Medicine, Mansoura University, Mansoura, 35516 Egypt; 2 Department of Pathology, Faculty of Veterinary Medicine, Mansoura University, Mansoura, Egypt

**Keywords:** Injectable platelet-rich fibrin, MSC-derived exosomes, Tendon regeneration, Superficial digital flexor tendonitis, Donkey, Diseases, Medical research, Rheumatology, Stem cells

## Abstract

Tendon injuries pose a significant challenge in equine medicine due to the inherently limited regenerative capacity of tendon tissue and the high likelihood of reinjury. Recently, injectable platelet-rich fibrin (I-PRF) and exosomes derived from mesenchymal stem cells (MSCs) have shown promise as regenerative treatments, offering potential benefits in matrix restoration and modulation of inflammatory responses. This study investigated the effects of I-PRF alone and in combination with MSC-derived exosomes on collagenase-induced superficial digital flexor (SDF) tendonitis in donkeys. Twenty-seven donkeys were randomly assigned to three treatment groups: saline (placebo), I-PRF, and PRF/exosome. Tendon healing was monitored over a 20-week period using clinical evaluation, ultrasonography, histology, immunohistochemistry, and biomechanical testing. At the study endpoint, the combined PRF/exosome group exhibited the greatest improvement in tendon consistency and weight-bearing ability compared to placebo (*P* < 0.001). Ultrasonographic findings were markedly better in the PRF/exosome group, even though tendon cross-sectional area (T-CSA) remained elevated across all treated groups, while it decreased significantly in the placebo group. Immunohistochemical analysis revealed the highest collagen type I and lowest type III levels in tendons treated with PRF/exosome. Biomechanical testing further demonstrated superior failure stress and strain in this group relative to placebo. Overall, these results indicate a potential synergistic effect of combining I-PRF with MSC-derived exosomes in promoting tendon repair. Nevertheless, additional long-term studies in naturally occurring tendon injuries are required to confirm these findings and assess their clinical relevance.

**Supplementary Information:**

The online version contains supplementary material available at 10.1038/s41598-026-44967-7.

## Introduction

Injuries to tendons, especially the superficial digital flexor tendon (SDFT), are common and often career-limiting in equines. Such injuries can account for as much as 72% of lost training days and contribute significantly to early retirement from athletic activity^1–3^. Equine SDF tendonitis not only affects horse performance but also imposes notable economic problems, with reported incidence rates ranging between 11% and 46% of all limb-related injuries^4,5^. Management of such injuries remains highly challenging, as tendon healing predominantly occurs through fibrotic scar formation rather than true regenerative^6^. Consequently, tendons seldom regain their native elasticity and mechanical integrity, which explains the high recurrence rates reaching nearly 80% following conventional therapies^7–9^.

Numerous treatment strategies have been suggested and continue to be developed for managing SDF tendonitis in horses. The ongoing emergence of these treatment approaches highlights both the complex pathophysiology of the condition and the continued difficulty in establishing clear evidence for their effectiveness^10^.

Recent progress in regenerative medicine has led to the development of various biologic treatments designed to enhance tendon healing, including mesenchymal stem cells and platelet-derived products (PDPs)^11,12^. Among PDPs, platelet-rich plasma (PRP) has been widely studied and has demonstrated promising outcomes in controlled experimental settings^13,14^. Yet, its therapeutic effectiveness in equine tendon repair continues to be debated in both experimental studies and clinical trials^10^. Two systematic reviews, including thirty studies, found that PRP improves lameness, tissue healing, and return-to-competition rates^15,16^. Conversely, a recent meta-analysis encompassing fifteen studies reported insufficient proof to support a significant contribution of PRP to tendon healing in equines^17^.

As a second-generation platelet concentrate, platelet-rich fibrin (PRF) provides distinct advantages compared with PRP. Its preparation is straightforward, requiring only autologous blood and simple stall-side handling. Unlike PRP, which releases growth factors for a limited period of around four days, PRF maintains a higher and more sustained release for up to two weeks^18,19^. Additionally, the fibrin-rich structure of PRF supports cell migration and provides a natural scaffold, without containing chemical additives that could potentially compromise its therapeutic effects^20^. Unlike the traditional clotted form, the liquid variant, known as injectable PRF (I-PRF), allows for direct administration into the tendon tissue. Although PRF has not yet been widely investigated in the management of equine SDF tendonitis, its application in other tendinopathies has yielded variable results^21–24^.

Although these biologics possess regenerative potential, their clinical outcomes in horses remain inconsistent, and complete regenerative repair is frequently not attained^10^. One of the emerging explanations for this inconsistency is inadequate resolution of the inflammatory response during tendon repair^25–27^. Prolonged or unresolved inflammation can delay progression to the reparative phase and may also reduce the effectiveness of regenerative treatments^28,29^. The initial phase of tendon repair is characterized by inflammation and cellular proliferation that, when inadequately regulated, may hasten tendon fiber deterioration. Concurrently, imbalanced collagen production promotes the predominance of mechanically inferior type III collagen over type I, ultimately giving rise to structurally compromised scar tissue^30^.

Understanding and precisely modulating inflammation has become central to improving treatment outcomes and promoting true regeneration^31^. Tendon stem cells and immune cells are essential regulators of tendon healing, particularly through their control of inflammation and coordination of repair^32,33^. Tendon stem/progenitor cells respond to inflammatory cytokines like IL-1β by releasing anti-inflammatory factors such as IL-10 and TIMP-3, helping to limit excessive inflammation and support tissue remodeling^34^. In parallel, immune cells, especially macrophages, transition from pro- to anti-inflammatory states, contributing to resolution and repair^35,36^. The interaction between these cell types is crucial in determining whether healing leads to regeneration or chronic degeneration^32^.

MSC-derived exosomes are gaining attention as a promising cell-free therapeutic approach for tendon healing, particularly through enhancing intercellular communication and modulating key signaling pathways involved in inflammation and regeneration^37^ and their ability to influence macrophage polarization^38,39^. Several experimental model studies have demonstrated that MSC-derived exosomes control inflammation and enhance tendon repair^37,40–42^. These exosomes activate main signaling pathways including PI3K/AKT and MAPK/ERK1/2, reducing apoptosis^42^, regulating the balance between ECM synthesis and degradation, and contributing to a more regenerative microenvironment^43,44^. Additionally, they modulate the immune response by downregulation of pro-inflammatory cytokines and upregulation of anti-inflammatory markers, ultimately reducing scar formation and improving biomechanical properties^45^. Additionally, MSC-derived exosomes contribute to tendon healing through the shift of macrophages toward a phenotype that supports tissue regeneration^39^. Furthermore, combining MSC-derived exosomes with fibrin scaffolds has demonstrated superior tendon healing outcomes by enhancing histological scores, upregulating tenogenic markers, and improving biomechanical properties, highlighting the synergistic potential of this approach in tendon regeneration^44^. 

Since tendonitis encompasses both inflammatory dysregulation and tissue degeneration, an intralesional therapeutic strategy that combines immunomodulatory and regenerative modalities could result in improved outcomes. This study investigates the effectiveness of PRF, either individually or combined with MSC-derived exosomes, in a donkey model of SDF tendonitis. It is hypothesized that MSC-derived exosomes may attenuate excessive inflammation while augmenting the regenerative capacity of PRF by facilitating more effective intercellular communication and signaling.

## Materials and methods

### Animals, ethics endorsement, and consent for participation

The Medical Research Ethics Committee of the Faculty of Veterinary Medicine, Mansoura University, approved this prospective, blinded, placebo-controlled experimental study (Code No: Ph.D./101). All procedures involving animals were carried out following institutional guidelines and national regulations for the care and use of laboratory animals. The study design and reporting adhered to the ARRIVE guidelines. Thirty-three clinically healthy male donkeys (36–48 ± 6.51 months old; 170 ± 11.68 kg) were obtained from the Faculty of Veterinary Medicine farm, Mansoura University, and housed in isolation stables at the Mansoura Veterinary Teaching Hospital, Egypt.

Before enrollment, all donkeys underwent clinical and ultrasonographic evaluations to confirm soundness. Clinical assessment included general and orthopedic physical examinations, with palpation of the tendons and evaluation of gait at a walk and trot in straight lines and circles on a hard surface. Ultrasonographic examination of the SDFTs was performed bilaterally to assess echogenicity and fiber alignment, ensuring the absence of pre-existing tendon pathology. Donkeys exhibiting lameness, swelling, heat, pain on palpation, or ultrasonographic abnormalities were excluded from the study.

Animals were kept under constant conditions and received a balanced diet of chopped wheat straw, bran, and whole corn. One week before the experiment, donkeys were dewormed with doramectin (1 ml/50 kg BW, IM; Dectomax, Zoetis, USA).

### Study design

**Fig. 1:**
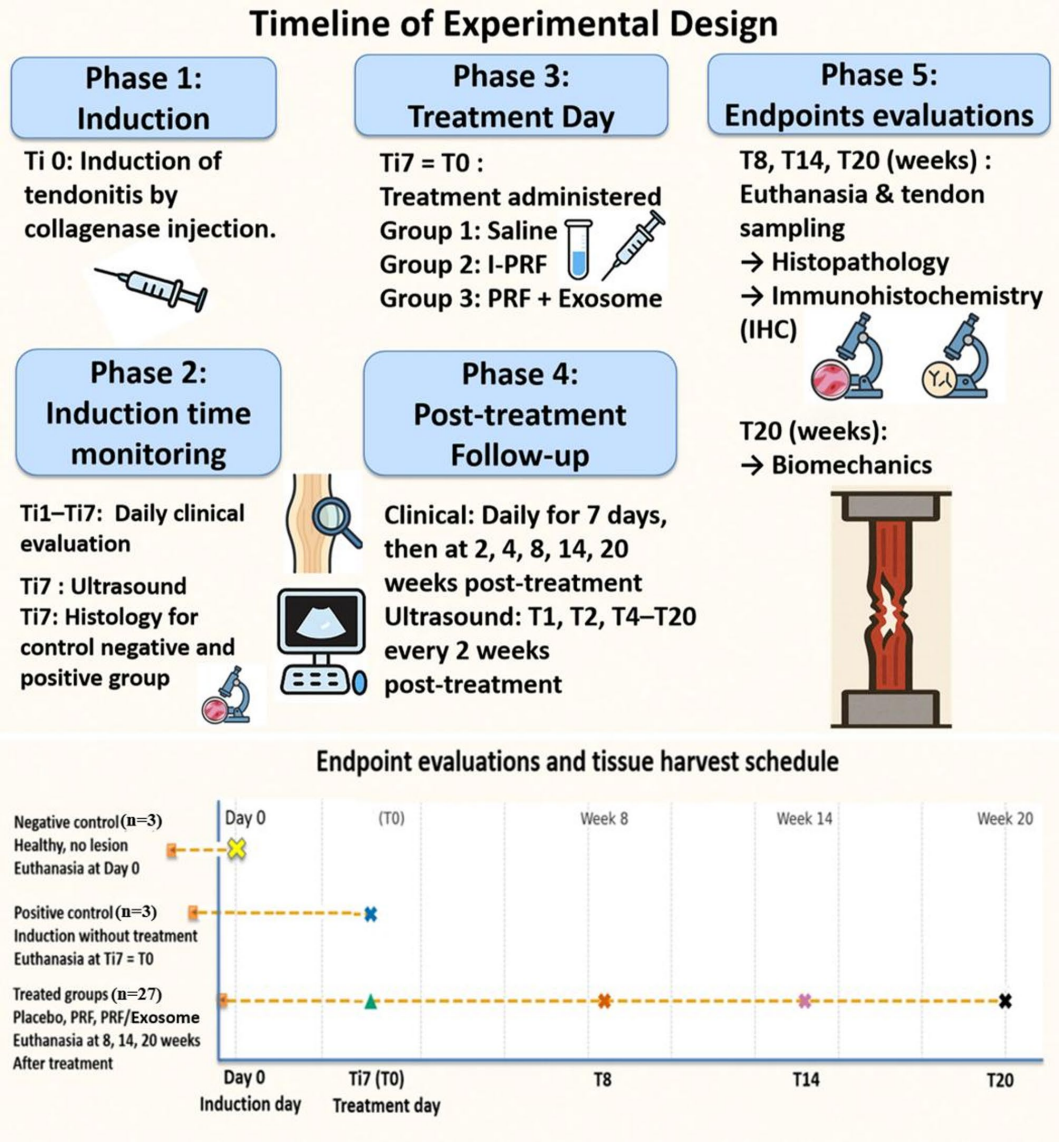
The time diagram illustrates the sequential phases of the experimental design.

SDF tendonitis was induced in both forelimbs of 30 donkeys at the mid-metacarpal region. After seven days, 27 donkeys were randomly assigned to one of three treatment groups (*n* = 9 each) using a computer-generated randomization sequence: the Placebo group, treated with saline; the PRF group, treated with I-PRF; and the PRF/Exosome group, treated with I-PRF and MSCs-derived exosomes. The remaining three donkeys (6 limbs) were retained as untreated tendonitis models (positive controls), while additional three healthy animals (6 limbs) were maintained as negative controls. Tendon healing was assessed through clinical, ultrasonographic, histopathological, immunohistochemical (IHC), and biomechanical evaluations at specific time points, as detailed later, with six forelimbs (*n* = 6) evaluated per group at each time point (Fig. 1).

### SDF tendonitis induction

The SDF tendonitis induction was accomplished under light general anesthesia using a modified previously described technique^46^. Donkeys received intravenous sedation with acepromazine (0.05 mg/kg, Castran, 15 mg/mL) followed 20 min later by xylazine HCl (1.1 mg/kg, Xyla-Ject, 20 mg/mL). Subsequently, butorphanol (0.05 mg/kg, Torbugesic 10 mg/mL) was administered. General anesthesia was induced using IV injection of thiopental (Anapental 500 mg, Corden Pharma, Egypt) at a dose rate of 2 mg/kg BW. On a lateral recumbency, the metacarpal (MC) regions of both limbs were aseptically prepared and scraped using povidone iodine. Under ultrasonographic guidance, an insulin needle was inserted laterally into the central SDFT at the mid-metacarpal region (midpoint between the distal end of the accessory carpal bone and the highest of the proximal sesamoid bones). A core lesion was then created via a sole injection of 2.5 mg collagenase (125 CDU/mg; Sigma, St. Louis, MO, USA) diluted in 0.3 mL saline. A pressure bandage was applied for 24 h post-injection. A three-day course of intramuscular procaine penicillin G (20,000 IU/kg SID) was administered, beginning one day before tendonitis induction and continuing for two days thereafter. Additionally, all animals administered systemic Flunixin meglumine (Flamicure 5%, Pharma Swede, Egypt) at a dose of 1.1 mg/kg once daily for three consecutive days starting immediately after collagenase injection, to control post-induction pain. The donkeys were stall rested for 24 h post-induction, then subjected to daily 5–15 min walking sessions for 7 days to simulate the mechanical load and stress associated with naturally occurring injuries.

### Autologous injectable PRF (I-PRF) preparation

Briefly, 4 ml of venous blood was drawn into a sterile plain plastic tube and immediately subjected to centrifugation at 700 rpm for three minutes (22 g RCF) using a rotor set at a 45° angle with a 40 mm radius. This process resulted in the separation of an upper I-PRF fraction from the underlying red blood cell layer, and the obtained I-PRF was administered within 5–10 min to prevent clotting^47^.

### Mesenchymal stem cell-derived exosome (MSC-derived Exosome) preparation

Exosomes were prepared immediately before treatment by reconstituting a 3 gm allogenic lyophilized powder (ExSommé, Bioluga^®^ Canada) in 2 ml of the supplied excipient, followed by the addition of 1 ml of sterile distilled water. The mixture was gently mixed until a clear and homogeneous injectable solution was obtained. According to the manufacturer, ExSommé^®^ contains lyophilized exosome concentrates derived from 6 × 10⁶ bone marrow-derived MSCs, with a protein concentration of 500 µg/ml (± 10%) as determined by BCA protein assay. Manufacturer-provided characterization indicated a mean particle size of approximately 130 nm measured by dynamic light scattering, typical cup-shaped vesicle morphology observed by transmission electron microscopy, and high expression (> 95%) of canonical exosomal markers CD63, CD81, and CD9.

### Treatment, follow-up, and controlled exercise

On the 7th day post-collagenase injection (Ti7 = T0), local injection of the treatment inoculum was accomplished on a lateral recumbency, after sedation and induction of thiopental anesthesia, and under complete aseptic preparation of the metacarpal regions of both forelimbs. Based on group allocation, the treatment inoculum of 3 mL volume contained either 3 mL of normal saline solution (placebo group), 1.5 mL of I-PRF + 1.5 mL saline (PRF group), or 1.5 mL I-PRF + 1.5 mL exosomes (PRF/Exosome group) and was administered using an 18-gauge needle under ultrasonographic guidance. The injection was distributed across three sites: intralesional [at the maximum injured zone (MIZ)], 0.5 cm above, and 0.5 cm below it. Following treatment, a pressure bandage was subsequently applied to the metacarpal region for one day. All animals were subjected to a progressively increasing exercise program, as previously described^48^. After a 3-day rest period following treatment, the exercise program commenced on day 4 with a 5-minute walking session per day. Walking time was extended at a rate of five minutes each week until reaching 30 min per day by week 7. Starting from week 8, the program incorporated trotting, beginning with 2 min of trotting per day and gradually increasing by 2-minute increments every two weeks.

### Evaluations of tendon healing

#### Clinical evaluation

Animals were carefully examined for degree of lameness, pain to pressure, hotness upon palpation, and presence of discomfort daily through the induction days (Ti1-Ti7) **(**Tables 1 and 2**)**. Following treatment, these evaluations were continued daily for the initial week, and subsequently at 2, 4, 8, 14, and 20 weeks post-treatment. Lameness scores were assessed in accordance with the modified scores by Riex et al.^49^. Pain and hotness were semi-quantitatively scored from 0 to 3, where 0 represented normal findings, 1 indicated mild symptoms, 2 denoted moderate manifestations, and 3 signified severe signs^48^. The donkeys were examined for the presence of discomfort using the modified donkey chronic pain scale^50^. Total discomfort scores ranged from 0 for comfort, 1–3 for mild discomfort, 4–6 for moderate discomfort, and 7–9 for severe discomfort.

**Table 1:** Clinical assessment scores for tendon healing^48,49^.

Score	Description
Heat	
0	Normal findings
1	Mild increase
2	Moderate increase
3	Severe increase
Pain	
0	Normal findings
1	Mild reaction
2	Moderate reaction
3	Severe reaction
Modified clinical score assessment for lameness	
0	Normal gait
1	Mild, intermittent lameness or difficult to observe, regardless of surface
2	Mild lameness, intermittent at a walk but consistently present under certain conditions (e.g., hard surface, weight-bearing)
3	Mildly abnormal gait and/or stiff walk
4	Reluctance to move when motivated/severe lameness at a walk
5	No movement or lying down; limb barely touches the ground (minimal weight-bearing or resting limb in flexion posture)
Tendon shape upon palpation	
0	Smooth, uniform normal thickening
1	Minimal irregularity or slight thickening
2	Notable irregularity
3	severe irregularity with a soft consistency
4	Hard nodular area
Intensifying weight bearing (static examination)	
0	Even weight distribution
1	Slight reduction in weight-bearing, slight preference for the opposite limb
2	Notable reduction in weight-bearing, notable preference for the opposite limb
3	Notable offloading of the limb

**Table 2:** Discomfort score according to a modified donkey pain scale^50^.

General appearance	
0	Alert and/or is interacting with a mate/group.
1	Mildly depressed and/or decreased interaction with mate/group.
2	Moderately depressed and/or aggressive, or no reaction with mate/group.
3	Severely depressed.
Standing Posture score	
0	Quietly standing and/or one hind leg resting.
1	Slightly tucked up abdomen and/or mild weight shifting.
2	Extremely tucked up abdomen and/or hunched back and/or stretching limbs/body and/or mild muscle tremors.
3	Sits on hind quarters and/or extreme muscle tremors.
Resting posture (lying down -Excluding Auto Grooming)	
0	Does not lie down or rest lying down.
1	Attempts to lie down or is lying down < 50% of the time.
2	Lying down > 50% of the time.
3	Lies down in an abnormal position: on its side with stretched limbs or on its back, and/or is repeatedly rolling.
0–9	Total discomfort score

Additionally, tendon shape and weight-bearing ability were examined at T0 and T20. To assess static load-bearing capacity, a controlled load equal to 30% of the animal’s body weight was applied. All evaluations were independently and blindly performed by two clinicians to ensure objective assessment.

#### Ultrasonographic evaluation

All tendons were evaluated using B-mode ultrasonography before collagenase injection (N), on day 7 post-collagenase injection, which also marked the start of treatment (Ti7 = T0), then at 1 week post-treatment (T1), at 2 week post-treatment (T2), and subsequently at two weeks intervals up to 20 weeks (T2–T20).This was carried out with a 7.5–10 MHz linear scanner in both longitudinal and cross-sectional planes.

Fiber echogenicity score (FES) was classified into four grades: grade 0 indicated normal echogenicity, grade 1 represented hypoechogenicity, grade 2 corresponded to heterogeneous or mixed echogenicity, and grade 3 denoted anechoic areas. Fiber alignment score (FAS) was determined based on the assessed proportion of parallel fibers as score 0 when more than 75% of fibers were parallel, score 1 when 50–74% were parallel, score 2 when 25–49% were parallel, and score 3 when less than 25% demonstrated parallel alignment (Table S1)^48^.

At the level of the MIZ, both the T-CSA and the L-CSA were quantified (mm²) using transverse sonograms. The proportion of lesion (Lesion %) was subsequently calculated by dividing the lesion cross-sectional area (L-CSA) by the total tendon cross-sectional area (T-CSA) and multiplying the result by 100^51^. Because significant intergroup variations were detected in baseline (T0) values of Lesion% and T-CSA, relative changes were assessed longitudinally within each group. For this purpose, baseline values were normalized to 1 (T0/T0), and measurements recorded at subsequent time points were normalized by dividing them by their respective baseline (T0) values^52^. US evaluations were performed by M.N., whereas image analyses were carried out by an independent radiologist blinded to treatment allocation. Each variable was measured in triplicate, and the mean of these values was then calculated and used for the subsequent statistical analysis.

### Tissue harvest

At the scheduled time points of T8, T14, and T20 weeks post-treatment, donkeys were sedated via IV injection of xylazine HCl at a dose of 2 mg/kg. Euthanasia was then achieved by rapid IV injection of a pentobarbital overdose^51^. At T8 & T14, the SDFTs were cautiously separated from the surrounding tissues. For histological and IHC evaluations, 2 cm tendon samples were harvested, including the central lesion site as well as adjacent proximal and distal healthy tissue. At T20, 7 cm-long SDFT specimens were harvested and longitudinally sectioned into two halves. One-half was submitted for biomechanical testing. In parallel, a 2 cm segment was excised from the second half and processed for histological and IHC analysis, following the same protocol as in earlier time points. Additionally, donkeys in the control positive group were euthanized on day 7 following collagenase injection to represent the baseline lesion state (T0) for histopathological evaluation. Meanwhile, control negative animals, which did not receive any tendon injury or treatment, served as the source of normal tendon specimens.

#### Histopathological evaluation

In weeks 8, 14, and 20, collected SDFT samples were processed following the routine histopathological preparation protocol. For histological assessment, three randomly selected sections from each sample were evaluated in a blinded manner by a pathologist. A modified semi-quantitative multiparametric scoring system was employed, as conducted in Table S2^53^. This scoring system involved the quantitative evaluation of six parameters, including: fiber structure, fiber arrangement, rounding of the nuclei, inflammatory cell infiltration, increased vascularity, and cell density. Each parameter was graded on a scale of 0 to 3, where 0 indicated normal arrangement, 1 represented mild abnormalities, 2 moderate tendon abnormalities, and 3severe tendon abnormalities.

#### Immunohistochemistry (IHC)

IHC was conducted to assess the relative proportions of collagen types I & III in tendon Sect. (5 μm thick) fixed on adhesion slides (Thermo Scientific, Fischer Scientific, UK) and processed following standard deparaffinization and rehydration protocols^48^.

For collagen type I staining, the sections underwent enzymatic antigen retrieval using Pronase and Hyaluronidase to unmask collagen epitopes embedded in dense matrix regions. Non-specific sites were blocked with goat serum, and the samples were then incubated with a primary rabbit polyclonal antibody against type I collagen. Detection was achieved with an HRP-conjugated secondary antibody and DAB chromogen, producing a brown signal at positive sites. Nuclei were counterstained with hematoxylin before dehydration and mounting.

Collagen type III staining employed a heat-induced antigen retrieval method using EDTA buffer, followed by suppression of endogenous peroxidase activity. After blocking with goat serum, sections were exposed to a primary rabbit polyclonal anti-collagen type III antibody. Signal amplification was accomplished using a biotinylated secondary antibody in conjunction with an ABC-HRP complex, and visualization was performed with DAB as described for type I.

All rinsing steps were carried out with phosphate-buffered saline, ensuring removal of unbound reagents between incubations. This approach enabled clear differentiation and quantification of the two collagen types within the tendon matrix.

For quantitative analysis, high-resolution digital images of selected regions of interest (ROIs) were extracted at magnifications up to ×40 and processed using ImageJ software (FIJI, National Institutes of Health, USA). Color deconvolution was applied to isolate the brown DAB chromogen signal from the hematoxylin counterstain, resulting in a DAB-specific monochrome image. A threshold was applied to highlight positively stained areas. The image was then binarized, and watershed segmentation was used to separate overlapping elements. Within each ROI, the DAB-positive area was quantified using frequency analysis, and the area fraction (percentage of positive staining relative to the selected area) was calculated. All measurements were performed in at least three representative ROIs per section, and the results were expressed as mean ± SEM^54^.

#### Biomechanical examinations

Immediately after collection, specimens were preserved in phosphate-buffered saline at − 20 °C, taking care to avoid repeated freeze-thaw cycles. Prior to testing, the samples were gradually brought to room temperature to allow complete thawing. The 7 cm-long SDFT specimens were loaded into a tensile testing device (Model LRX, Lloyd Instruments Ltd, UK), with a jaw separation of 4.2 cm. Sandpaper was placed between the sample ends and the clamps to minimize slippage during testing. After stabilization, the midsection’s width and depth were recorded in the transverse plane with a digital caliper to determine the cross-sectional area. A small preload of 5 N was applied for five cycles at 1 Hz to remove specimen laxity. Each specimen was then subjected to uniaxial tension until failure using a 200 kgf load cell at a steady crosshead speed of 10 mm/min. The peak force recorded before rupture was taken as the failure load (N), while failure stress (MPa) was calculated by dividing this load by the sample’s cross-sectional area. The strain (%) was determined by expressing the elongation at the point of failure relative to the specimen’s initial length^55^.

#### Statistical analysis

Statistical evaluation was performed in SPSS v28 (IBM Corp., Armonk, NY). Data distribution was evaluated with the Shapiro–Wilk test. Normally distributed variables are summarized as mean (SD), whereas ordinal data are reported as median (min–max). Parametric variables were evaluated with two-way ANOVA followed by Tukey adjustment. To further control for Type I errors across groups and time points, post-hoc analyses were adjusted using the Bonferroni method. Paired t-tests compared T-CSA at T20 with pre-induction (Normal) and baseline (T0). Groups were compared for change from baseline (T0) for lesion percentage and T-CSA. Nonparametric data were analyzed using Kruskal–Wallis (between-group), Friedman (within-group over time), and Wilcoxon signed-rank tests (T0 vs. T20). Statistical significance was defined as *P* ≤ 0.05, and all graphs and visual representations were generated using GraphPad Prism version 8.4.3.

## Results

### Clinical findings

During the tendonitis induction period, pain and heat scores peaked within the first two days post-collagenase injection, with median scores of 3 (3–3), displaying severe pain reactions upon palpation and a marked increase in local temperature. By the 3rd day, a significant reduction was observed compared to day 1 (*P* = 0.027), with scores declining to 2 (1–2), where animals exhibited moderate pain and heat responses. By the 4th day, a further significant decrease was noted compared to day 3 (*P* = 0.016), with the score reaching 1 (0–1), representing mild heat and pain responses. By the 7th day, pain and heat responses had significantly diminished to 0 (0–1) compared to day 4 (*P* = 0.006, *r* = 0.65, 95% CI 0.30–0.85), reflecting normal findings in most animals, and only a few animals showed a mild response.

Similarly, all animals exhibited a severe lameness during the first two days following collagenase injection, with a median score of [S = 5 (5–5)]. Where animals were either in a recumbent position or maintained their limbs in a flexed posture while standing, lightly touching the ground with minimal weight-bearing. By the 4th day, a significant reduction in the lameness score was observed compared to day 1 (*P* = 0.000), decreasing to 2 (2–3), where animals exhibited either a mild abnormal gait (S = 3) or a consistently difficult observable lameness (S = 2). Further significant improvement was noted by the 6th day (*P* = 0.039), with the lameness score decreasing to 1 (1–2), representing intermittently difficult-to-detect lameness. By the 7th day, the lameness score had significantly reduced to 0 (0–1), representing a return to normal gait (S = 0) in most animals, with only a few exhibiting intermittent difficult-to-detect lameness (S = 1), with significant differences compared to day 6 (*P* = 0.034) and non-significance difference compared to the normal value (*P* = 0.576, *r* = 0.19, 95% CI − 0.15–0.50).

Concerning discomfort assessments, the general appearance score revealed a mild to moderate decrease in alertness and group interaction 1 (1–2) during the first two days following collagenase injection. By the 4th day, a significant improvement was observed compared to day 1 (*P* = 0.006), with the score reducing to 1 (0–1), where most animals exhibited a mild decrease in alertness and interaction with their group (S = 1), while others had returned to normal activity (S = 0). By the 6th day, the general appearance score had significantly returned to 0 [S = 0 (0–0)], representing full activity, with all animals being alert and interacting normally, with no significant difference compared to day 5 (*P* = 0.276).

A mild alteration in the standing posture score was observed during the first two days post-collagenase injection [S = 1 (1–1)], where animals were observed in mild weight shifting. By the 4th day, a significant improvement was noted compared to day 1 (*P* = 0.000), as all animals returned to a normal standing posture [S = 0 (0–0].

A mild alteration in resting posture was observed during the first three days post-collagenase injection, as animals either attempted recumbency or limited time in a lying position (S = 1). By the 5th day, a significant improvement was noted compared to day 1 (*P* = 0.000), with the score returning to the normal value [S = 0 (0–0)].

**Fig. 2:**
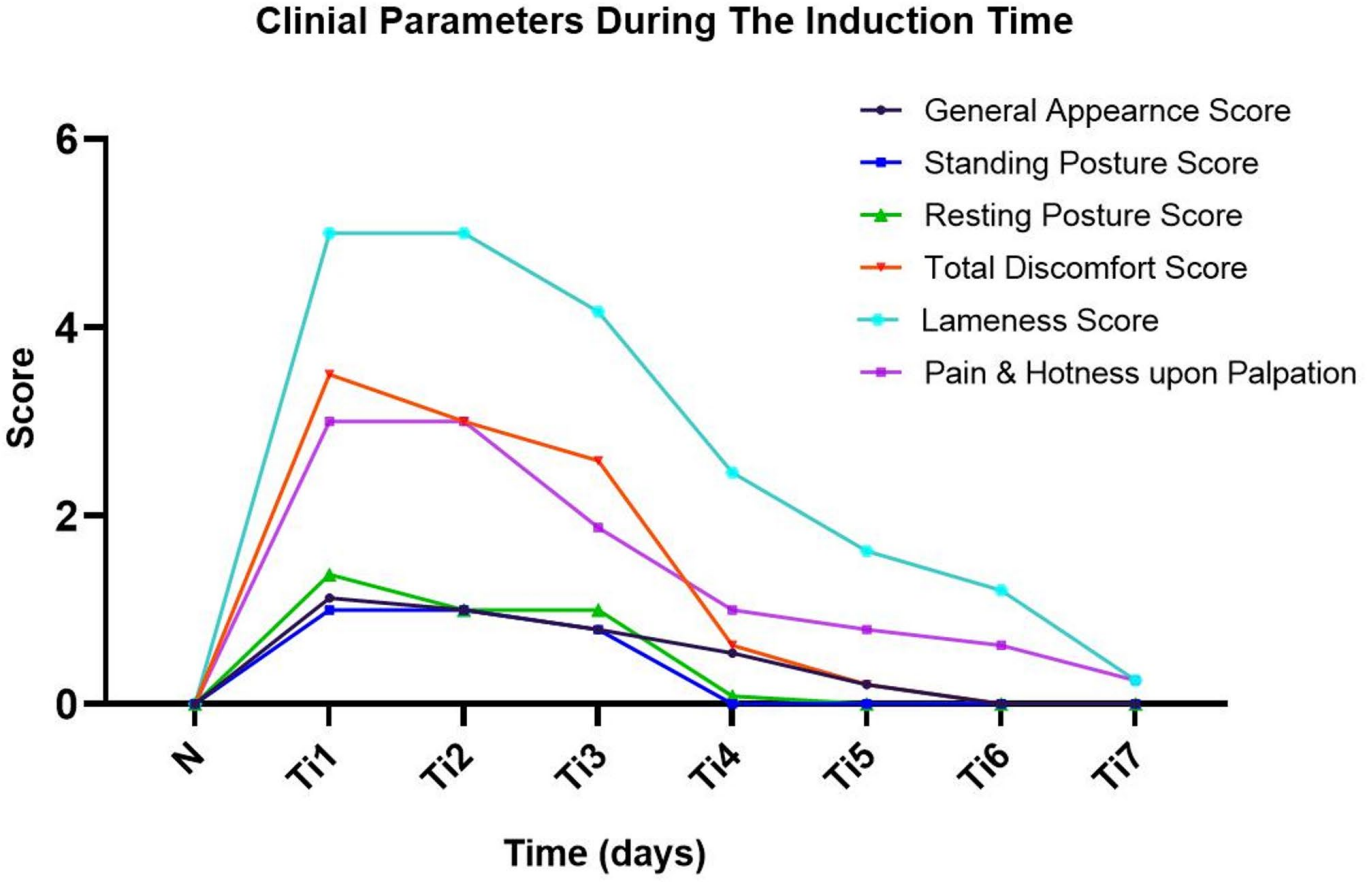
Showing the clinical evaluation parameters during the tendonitis induction period (Ti1–Ti7) in comparison to the normal baseline value (N).

Finally, the overall discomfort score exhibited a mild increase in the first three days post collagenase injection [S = 3 (3–5)]. By the 5th day, a significant reduction was observed compared to day 1 (*P* = 0.000), with scores decreasing to 0 (0–1), where only mild signs of discomfort persisted in a few animals (*n* = 13/30). By the 6th day, the total discomfort score had further decreased to score 0 (0–0), indicating a complete return to normal behavior, with a significant difference compared to day 1 (*P* = 0.000, *r* = 0.85, 95% CI 0.70–0.92) and a non-significant difference compared to the normal value (*P* = 1.000, *r* = 0.0, 95% CI − 0.05–0.05) (Table S3, Fig. 2).

Regardless of the biomaterials used for treatment, there was no significant difference in the clinical observation parameters between all groups (*P* > 0.05) at any time point after treatment, except for intensifying weight-bearing and tendon shape scores at T20. Additionally, Time had no significant effect on these parameters in any group compared to T0 (*P* > 0.05), except for intensifying weight-bearing and tendon shape scores.

Regarding the pain and heat scores, the placebo and PRF/Exosome groups returned to normal scores by day 3, while the PRF group by day 4 post-treatment. In terms of lameness scores, donkeys in the PRF/Exosome group became sound by day 3 post-treatment, those in the PRF group by day 4, and those in the placebo group by day 7.

**Fig. 3:**
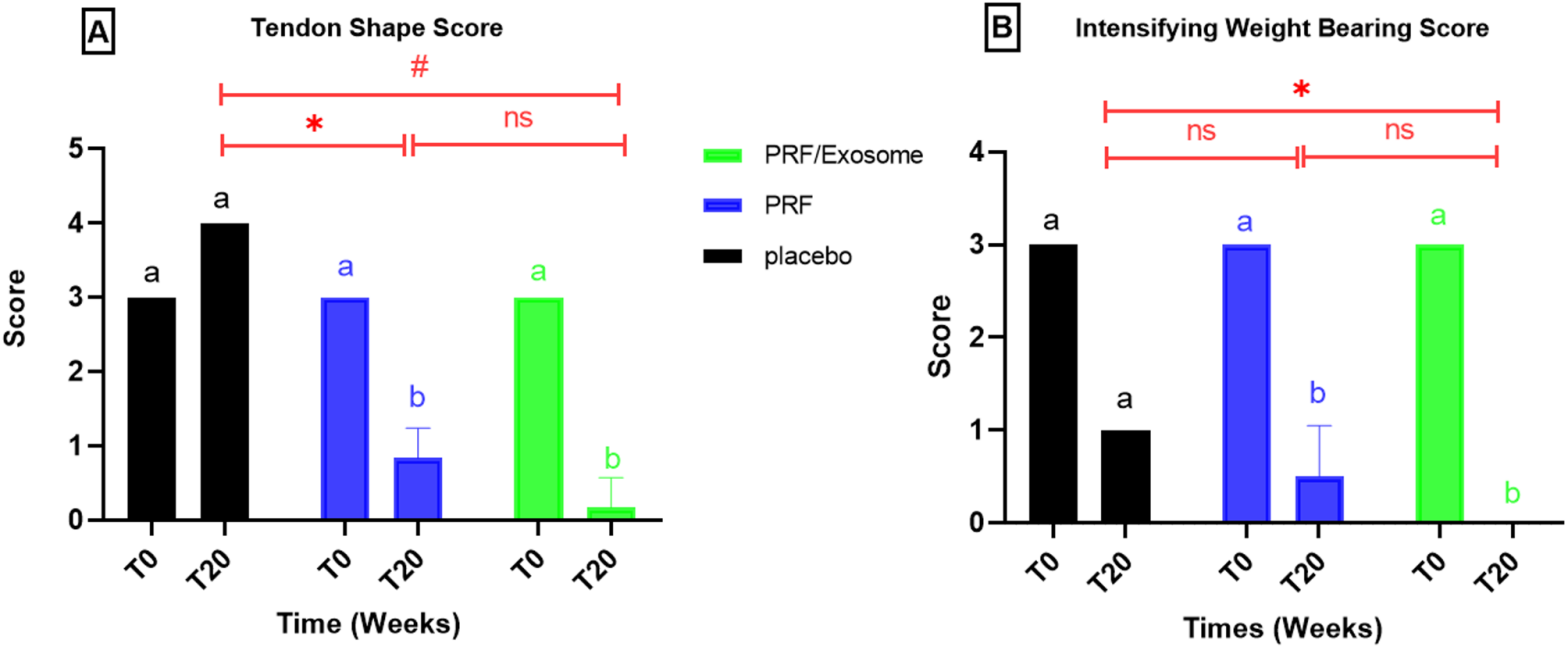
Showing the clinical evaluation of palpable shape of the tendons (**A**), and intensifying weight carrying score (**B**) in both placebo and treated groups at T0 & T20. Within-group comparisons between the two time points are indicated by colored horizontal lines: black lines for the Placebo group, blue lines for the PRF group, and green lines for the PRF/Exosome group. Between-group comparisons at time point T20 are indicated by red horizontal lines. Statistical significance is denoted as follows:* p < 0.05, ** p < 0.01, *** p < 0.001, & ns = not significant.

At T0, all groups exhibited an irregular tendon shape with soft consistency [S = 3(3–3)], with no significant differences between them (*P* = 1.000). By T20, the placebo group expressed hard nodular area in the tendon [S = 4(4–4)], while the PRF group showed minimal tendon thickening (S = 1 (0–1)), with a non-significant difference from normal values (*P* = 0.149). Additionally, the PRF/Exosome group demonstrated near-complete recovery at T20, with most donkeys (*n* = 7/9) achieving normal tendon shape and consistency [S = 0 (0–1)] with non-significant difference compared to normal (*P* = 0.773) (Table S4, Fig. 3A).

Regarding intensifying weight-bearing, all groups displayed notable offloading of the limb [S = 3 (3–3)] at T0, with no significant differences between them (*P* = 1.000). At T20, the placebo group showed only a slight reduction in weight-bearing ability [S = 1 (1–1)], with no significant difference from normal values (*P* = 0.083). Conversely, all PRF and PRF/Exosome groups achieved balanced weight distribution (S = 0), showing a significant improvement compared with baseline (*P* < 0.05). The PRF/Exosome group fully normalized weight-bearing ability (*P* = 1.000), whereas the PRF group exhibited a non-significant decrease relative to normal values (*P* = 0.386) (Table S4, Fig. 3B).

#### Ultrasonographic findings

The normal tendon cross-sectional areas (T-CSA), lesion cross-sectional areas (L-CSA), and the corresponding lesion percentages and the corresponding lesion sizes for each treatment group are summarized in Table S5.

During weeks 4 and 6 (T4 & T6), the lesion % in the placebo and PRF groups continued to rise significantly (*P* ≤ 0.05), reaching the highest level during this period, with no significant difference observed between the two groups (*P* > 0.05). In contrast, the PRF/Exosome group exhibited only a slight, non-significant increase in lesion % at T4 compared to T0 (*P* = 0.9952), followed by a gradual and steady decline by T6.

From week 8 onward, all groups exhibited a gradual decline in lesion %, though at varying rates. In the placebo group, the first drop below T0 was observed at T14, although this reduction was not statistically significant (*P* = 0.2065). A significant reduction in lesion % in the placebo group was first observed at T18 (*P* < 0.0001). In comparison, the PRF group showed its first significant reduction below T0 as early as T10, with a marked statistically significant difference compared to the placebo group (*P* < 0.0001). The PRF/Exosome group continued a steady decline after T6, with the first significant reduction below T0 occurring at T10 (*P* = 0.0493) and a highly significant decrease by T12 (*P* < 0.0001).

**Fig. 4:**
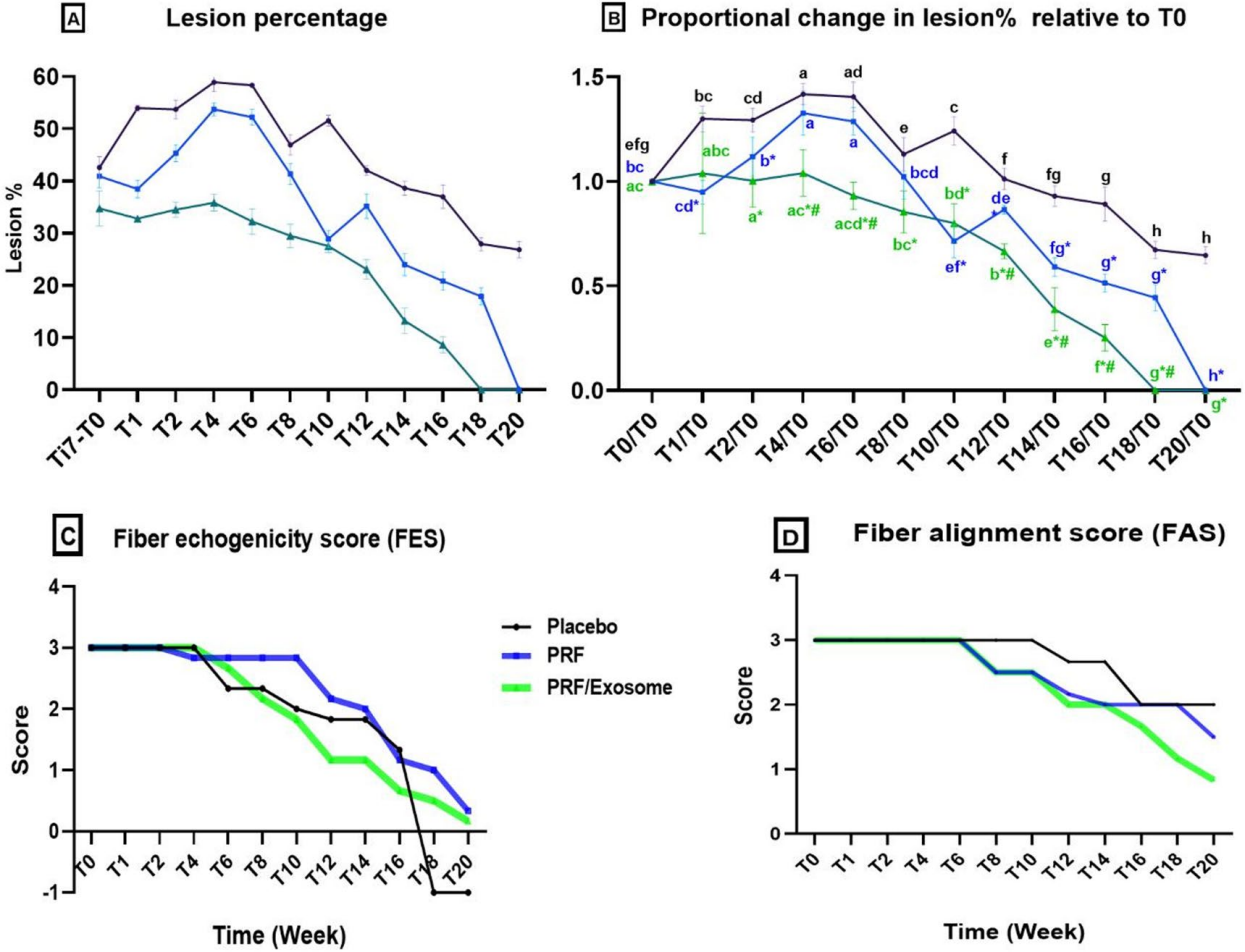
Showing the Lesion % (**A**), proportional change in lesion % over time relative to T0 (**B**), FES (**C**), and FAS (**D**) in both control and treated groups. Times with different small letters are significant in a group at *P* > 0.05. * There is a significant difference compared to the placebo group at the same time, at *p* < 0.05. **#** There is a significant difference compared to the PRF group at the same time, at *p* < 0.05.

The disappearance of lesions occurred at varying times across the treatment groups. In the placebo group, the anechoic core lesion had completely resolved by T18 and was replaced by hyperechoic dots. In the PRF group, complete lesion resolution was noted at T20. The PRF/Exosome group achieved complete lesion resolution by T18 (Table S6, Fig. 4A, B).

Concerning the tendon cross sectional area (T-CSA) during the first two weeks following treatment, all groups exhibited a significant increase (*P* < 0.05) except the PRF/Exosome group which showed only slight a non-significant increase (*P* = 0.981) compared to T0, with this increase showed a significantly lower value compared to the placebo (*P* = 0.0001) and PRF (*P* = 0.001) groups. After this initial phase, T-CSA values across all groups exhibited fluctuating patterns, alternating between increases and decreases. At T20, the placebo group showed a significant decrease in T-CSA (35.38 mm² ± 0.63) compared to T0 (*P* = 0.001). The PRF and PRF/Exosome groups showed consistently higher T-CSA values than T0 throughout the study, with a slight, non-significant decrease observed at T20 (Table S7). Compared to the normal tendon size, the T-CSA at T20 remained higher in all groups to varying degrees. The placebo group showed a slight increase of 13% (*P* < 0.0001). The PRF/Exosome group exhibited a mild increase of 25%, while the PRF group showed a marked increase of 34% (*P* < 0.0001) relative to the normal T-CSA.

Compared to T0, the FES showed a significant decrease for the first time at T10 across all groups, with varying scores: [S = 2 (2–2) in both the placebo (*P* = 0.031, *r* = 0.70, 95% CI 0.40–0.90) and PRF groups (*P* = 0.028, *r* = 0.73, 95% CI 0.42–0.91); and 2 (1–2) in the PRF/Exosome group (*P* = 0.037, *r* = 0.70, 95% CI 0.40–0.90). Nearly normal echogenicity was observed at T18 in the PRF/Exosome group [S = 0.5 (0–1)], and at T20 in the PRF group [S = 0 (0–1)]. In the placebo group, the lesion was significantly replaced by hyperechoic tissue at both T18 and T20 (*P* = 0.000). There was no significant difference in FES between the placebo group and both PRF and PRF/Exosome groups at most time points (*P* > 0.05) except at T18 and T20 (*P* < 0.05). The PRF/Exosome group showed a significant difference compared to the PRF group just at T8, 12, and T14 (*P* < 0.05), where it showed a more rapid improvement in FES at those time points (Table S8, Fig. 4C).

Regarding FAS, there was no significant difference between the placebo and PRF groups at all time points (*P* > 0.05). The PRF/Exosome group exhibited a significant improvement in FAS compared to the PRF and Placebo groups at T18 (*P* = 0.042, *r* = 0.47, 95% CI 0.05–0.70). Compared to T0, the FAS in the Placebo group showed a significant decrease for the first time at T16 (*P* = 0.004), representing fibers with 25–49% of the normal alignment pattern [S = 2(2–2)], and remained unchanged thereafter. This parameter decreased significantly in the PRF and PRF/Exosome groups earlier at T12 (*P* < 0.05). At T20, a non-significant decrease was observed in the PRF group [S = 1.5 (1–2)] compared to T12, and the PRF/Exosome group exhibited a significant decrease [S = 1(0–1)] compared to T12, representing fibers with 50–74% of the normal alignment pattern (Table S9, Fig. 4D).

#### Histopathological findings

Tendons from the control positive group (at day 7 post-collagenase injection; Ti7) displayed marked histological alterations, where the normal tendon structure was replaced by a dense inflammatory infiltrate composed predominantly of neutrophils, lymphocytes, and macrophages. Moreover, pronounced neovascularization was observed, accompanied by multifocal hemorrhages.

**Fig. 5:**
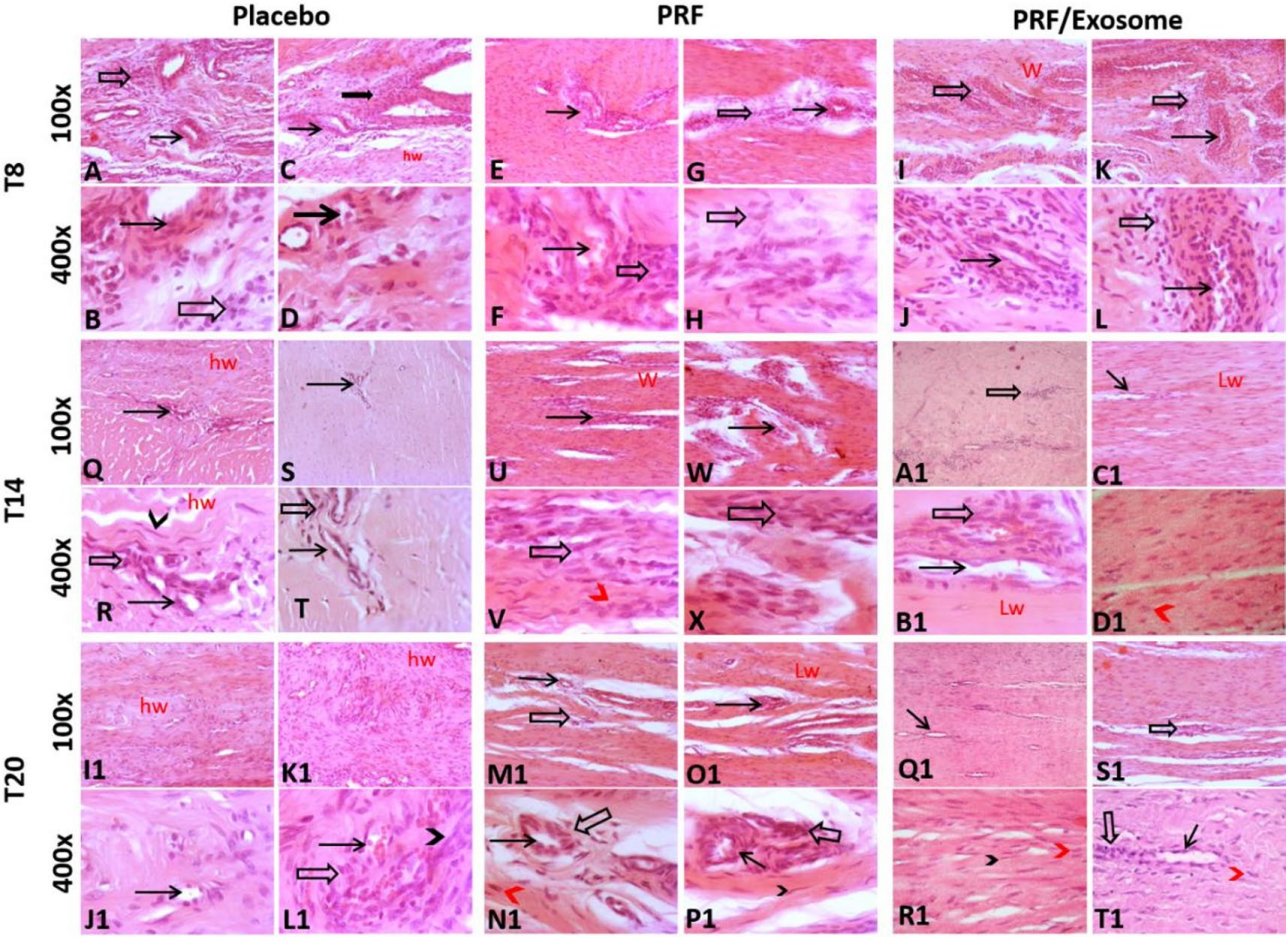
Representative photomicrograph of tendon from different treatment groups. AT T8 (**A**-**D**) Placebo group showing diffuse severe replacement of tendon tissue by many blood vessels surrounded by abundant inflammatory cells composed of many neutrophils, lymphocytes, and macrophages. (**E**-**H**) PRF group showing focal to coalescing inflamed fibrovascular tissue with newly formed blood vessels surrounded by many lymphocytes, macrophages, and immature round fibroblasts. (**I**-**L**) PRF/Exosome group showing disorganized tendon with severe replacement by many blood vessels surrounded by abundant lymphocytes, plasma cells, and rounded fibroblasts. AT T14 (**Q**-**T**) Placebo group showing highly wavy tendon with a focal few blood vessels surrounded by low numbers of inflammatory cells as lymphocytes, plasma cells, and mild numbers of spindle fibroblasts. (**U**-**X**) PRF group showing less organized wavy tendon fibers with multifocal angiogenesis, few extravasated RBCs with many rounded fibroblasts, and mild numbers of inflammatory cells as lymphocytes. A1:D1) PRF/Exosome group showing little wavy arranged tendon fibers with few focal blood vessels surrounded by mild to moderate numbers of extravasated RBCs, admixed with rounded fibroblasts and a few macrophages, lymphocytes. AT T20 I1:L1 Placebo group showing disorganized tendon fibers with few angiogenesis, scattered extravasated RBCS, haphazard rounded to spindle fibroblasts. M1:P1) PRF group showing mild focal to coalescing blood vessels surrounded by moderate numbers of spindle fibroblasts. Q1:T1) PRF/Exosome group showing little wavy bundles and a few regular arrangements of tendon fibers with a few focal aggregations of inflammatory cells and spindle fibroblasts. Thin arrow = neovascularization, thick arrow = inflammation with fibroblast proliferation (either rounded = red arrowhead or spindle = black arrowhead), w = wavy tendon arrangement, hw = highly wavy disorganized collagen, lw = little wavy. Image magnification=100x, 400x.

The administration of regenerative biologics to the SDFT induced notable histopathological improvements. Statistical analysis revealed significant differences among treatment groups in all histopathological assessment scores at T20. Moreover, time exerted a positive effect on improving these scores across the PRF/Exosome group. However, no significant temporal changes were observed in fiber arrangement, fiber structure, and cell density within the placebo group, nor in fiber arrangement, fiber structure, and nuclear morphology within the PRF group, where values remained statistically nonsignificant compared to T0 (*P* > 0.05) (Table 3; Fig. 5).

**Table 3:** Shows results of histopathological assessment scores between the different groups.

Group	Evaluation times					Friedman’s test		*P* value
	T0 (Control positive group)		T8	T14	T20			
Angiogenesis								
Placebo	3(3–3)^a^	3(3–3)^a^		1.5(1–2)^bc^	1(1–1)^c^		17.471	0.001
PRF	3(3–3)^a^	3(3–3)^a^		3(2–3)^a*^	2(1–2)^b*^		16.286	0.001
PRF/Exosome	3(3–3)^a^	3(2–3)^a^		1(1–2)^b#^	1(1–2)^b^		16.043	0.001
Kruskal-wallis	0.000	15.046		13.065	11.764			
P value	1.000	0.002		0.004	0.008			
Inflammatory cell infiltration								
Placebo	3(3–3)^a^	3(3–3)^a^		1.5(1–2)^b^	1(1–1)^b^	17.471		0.001
PRF	3(3–3)^a^	3(3–3)^a^		1.5(1–2)^b^	2(1–2)^b*^	17.694		0.001
PRF/Exosome	3(3–3)^a^	3(2–3)^a^		1(1–2)^b^	1(1–2)^b^	16.043		0.001
Kruskal-wallis	0.000	10.767		1.874	11.764			
P value	1.000	0.013		0.599	0.008			
Fiber structure								
Placebo	3(3–3)	3(3–3)		3(3–3)	3(3–3)	0.000		1.000
PRF	3(3–3)^a^	3(3–3)^a^		3(2–3)^a^	2.5(2–3)^a^	7.364		0.061
PRF/Exosome	3(3–3)^a^	3(3–3)^a^		2(2–3)^ab*^	1(1–2)^b*#^	16.680		0.001
Kruskal-wallis	0.000	0.000		16.560	19.943			
P value	1.000	1.000		0.001	0.000			
Fiber arrangement								
Placebo	3(3–3)	3(3–3)		3(3–3)	3(3–3)	0.000		1.000
PRF	3(3–3)^a^	3(3–3)^a^		2.5(2–3)^a^	2(2–3)^a^	8.053		0.045
PRF/Exosome	3(3–3)^a^	3(3–3)^a^		1.5(1–2)^b*#^	1(0–1)^b*#^	17.471		0.001
Kruskal-wallis	0.000	0.000		16.962	20.125			
P value	1.000	1.000		0.001	0.000			
Rounding of nuclei								
Placebo	3(3–3)^a^	3(3–3)^a^		3(2–3)^a^	1(0–1)^b^	16.286		0.001
PRF	3(3–3)^a^	3(3–3)^a^		3(2–3)^a^	2(1–3)^a*^	7.333		0.062
PRF/Exosome	3(3–3)^a^	3(3–3)^a^		2(1–2)^b*#^	1(0–2)^b^	17.583		0.001
Kruskal-wallis	0.000	0.000		12.230	9.922			
P value	1.000	1.000		0.007	0.019			
Cell density								
Placebo	3(3–3)^a^	3(3–3)^a^		3(3–3)^a^	3(2–3)^a^	6.000		0.112
PRF	3(3–3)^a^	3(3–3)^a^		2.5(2–3)^ab^	2(2–3)^b^	12.000		0.007
PRF/Exosome	3(3–3)^a^	3(3–3)^a^		1.5(1–2)^b*#^	1(1–2)^b*#^	16.846		0.001
Kruskal-wallis	0.000	0.000		16.962	17.084			
P value	1.000	1.000		0.001	0.001			
Total histological score								
Placebo	3.0 ± 0.0	3.00 ± 0.00		2.5 ± 0.77^b^	2.0 ± 1.09	-		-
PRF	3.0 ± 0.0^a^	3.00 ± 0.00^a^		2.5 ± 0.58^ab^	2.0 ± 0.20^b^	-		-
PRF/Exosome	3.0 ± 0.0^a^	3.00 ± 0.00^a^		1.5 ± 0.45^b*^	1.0 ± 0.00^b#^	-		-

Times with different superscript letters are significantly different at *p* < 0.05, df = 3

*significant difference compared to the placebo group in the same time at *p* < 0.05, df = 3

#significant difference compared to the PRF group in the same time at *p* < 0.05, df = 3

By T20, all groups showed a statistically significant reduction in inflammatory cell infiltration and angiogenesis compared to T0, although the extent of improvement varied (Placebo: *P* = 0.003, *r* = 0.97, 95% CI 0.60–0.99; PRF: *P* = 0.01, *r* = 0.83, 95% CI 0.50–0.96; PRF/Exosome: *P* = 0.007, *r* = 0.90, 95% CI 0.55–0.98). The placebo group recorded the lowest scores (S = 1 [1–1]), corresponding to minimal inflammatory cell infiltration and angiogenesis. The PRF/Exosome group also improved [S = 1 (1–2)] at T20, yet retained higher scores than the placebo group. In contrast, the PRF group showed the least improvement [S = 2 (1–2)], representing persistent moderate inflammation.

Concerning the fiber structure of the newly formed tissue at the AOI, the PRF/Exosome group exhibited mildly to moderately fragmented fibers (S = 1 [1–2]), with a significant improvement from T0 (*P* = 0.002, *r* = 1.03, 95% CI 0.65–1.0). In contrast, the PRF group displayed moderate to severe fragmentation (S = 2.5[2–3]), with a non-significant reduction compared to T0 (*P* = 0.061). The placebo group consistently showed severely fragmented fibers throughout the study period. Intergroup comparison at the end of the study revealed that the PRF/Exosome exhibited significantly better outcomes than the PRF group (*P* = 0.034, *r* = 0.50, 95% CI 0.05–0.75) and the placebo group (*P* = 0.003, *r* = 0.68, 95% CI 0.40–0.90).

Similarly, the PRF/Exosome group exhibited notable improvement in fiber arrangement at T20 compared to T0 (*P* = 0.003, *r* = 0.97, 95% CI 0.60–0.99), with scores reflecting slightly loose, wavy fibers (S = 1 [1–1]). The PRF group retained higher scores at T20, representing moderately loose to completely disorganized fiber architecture [S = 2 (2–3)]. The placebo group consistently showed severely disrupted fiber arrangement throughout the study period [S = 3 (3–3)], with no identifiable fiber pattern. At T20, the PRF/Exosome group demonstrated significantly improved scores compared to the PRF group (*P* = 0.021, *r* = 0.54, 95% CI 0.10–0.75) and the placebo group (*P* = 0.001, *r* = 0.73, 95% CI 0.40–0.90).

At T20, the PRF/Exosome group exhibited significantly improved nuclear morphology compared to T0 (*P* = 0.001, *r* = 1.03, 95% CI 0.65–1.0), with scores ranging from spindle-shaped to moderately rounded nuclei (S = 1 [0–2]), but did not differ significantly from the PRF group (*P* = 0.071). In contrast, the PRF group maintained higher scores (S = 2 [1–3]), representing the persistence of slightly to severely rounded nuclei, and differed significantly from the placebo group (*P* = 0.004, *r* = 0.68, 95% CI 0.40–0.90). The placebo group at T20 showed significant improvement from T0 (*P* = 0.004, *r* = 0.97, 95% CI 0.60–0.99), with lower scores (S = 1 [0–1]) representing predominantly spindle-shaped or slightly rounded nuclei, and no significant differences were observed when compared to the PRF/Exosome groups (*P* = 0.277).

Compared to the pattern of cell density observed at T0, a significant improvement was detected at T20 in the PRF/Exosome groups (*P* = 0.004, *r* = 0.97, 95% CI 0.60–0.99), with scores reflecting a slight to moderate increase in cellularity (S = 1 [1–2]). In contrast, both the PRF and placebo groups showed higher scores, demonstrating a moderate to severe increase in cell density (S = 2 [2–3] and 3 [2–3], respectively). Intergroup comparisons at T20 revealed significantly improved scores in the PRF/Exosome groups relative to both the PRF group (*P* = 0.017, *r* = 0.57, 95% CI 0.10–0.75) and the placebo group (*P* = 0.001, *r* = 0.73, 95% CI 0.40–0.90).

Across the study timeline, all groups showed a gradual improvement in total histological scores from T8 to T20. The PRF/Exosome group showed the most notable improvement (1.0 ± 0.00) at T20, significantly differing from the PRF group (*P* = 0.027, *r* = 0.52, 95% CI 0.05–0.75). In contrast, the PRF and placebo groups retained higher scores at T20 (2.0 ± 0.20 and 2.0 ± 1.09), with no significant difference between them (*P* = 0.603).

### Immunohistochemical findings

**Fig. 6:**
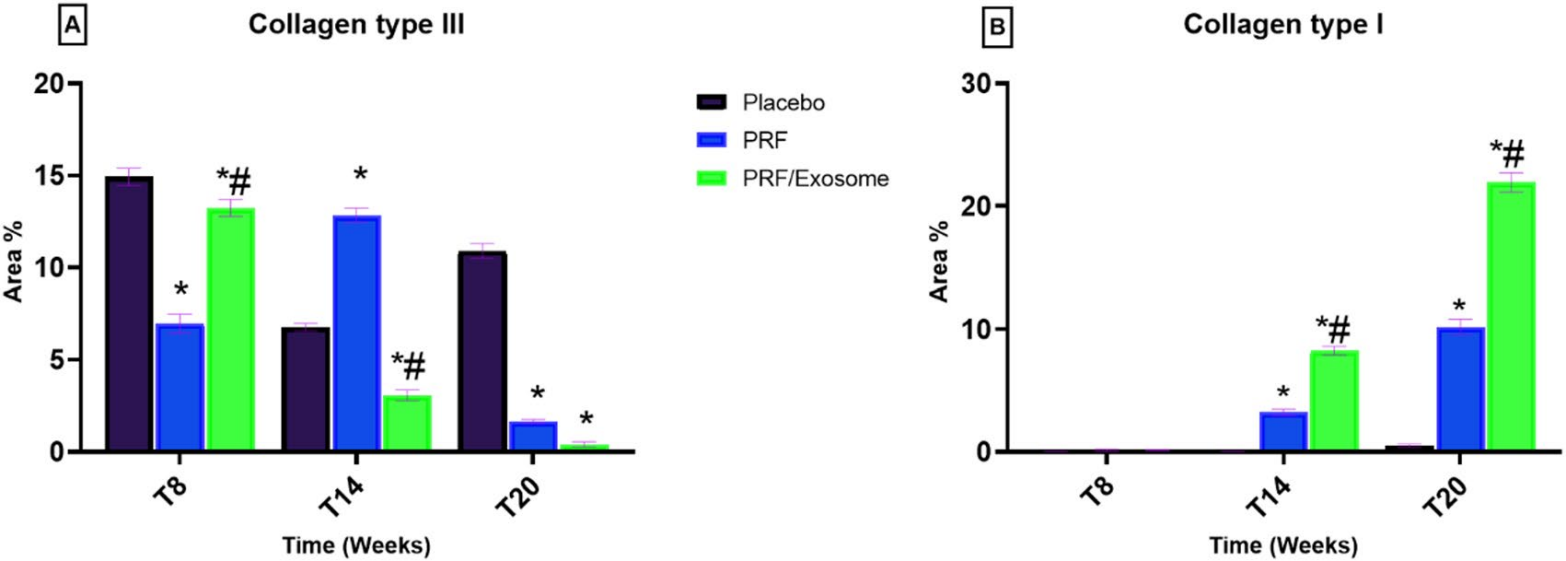
Immunohistochemical semi-quantitative analysis of collagen type III (**A**) and collagen type I (**B**) in SDFT sections at different time points across treatment groups.

Time exerted a consistent and significant effect in reducing collagen type III area percentage across all groups (*P* < 0.05). At T20, the PRF/Exosome group exhibited the lowest collagen III content (0.39 ± 0.16%), which was significantly lower than both the PRF group (1.65 ± 0.12%, *P* < 0.0001) and the placebo group (10.89 ± 0.45%, *P* < 0.0001). Nevertheless, all treated tendons still showed significantly higher collagen III levels at T20 compared to the normal values (Table S10, Fig. 6A).

Time had a significant effect in increasing the collagen type I area percentage across all treatment groups (*P* < 0.05). At T8, the placebo group showed minimal collagen type I content (0.022 ± 0.005%), with no significant change at T14 (0.020 ± 0.014%, *P* = 0.985). In contrast, the PRF (0.92 ± 0.11%, *P* = 0.0056) and PRF/Exosome (0.13 ± 0.05%, *P* = 0.0002) groups demonstrated significantly higher levels compared to the placebo group. By T20, collagen type I markedly increased in all biomaterials treated groups, with significantly higher values recorded in the PRF/Exosome (21.93 ± 0.79%, *P* < 0.0001) group, followed by the PRF group (10.17 ± 0.64%, *P* = 0.0024), in comparison to the placebo group (0.494 ± 0.122%) (Table S10, Fig. 6B). All groups exhibited significantly lower collagen type I values compared to the normal tendon baseline (*P* < 0.05).

### Biomechanical finding

**Fig. 7:**
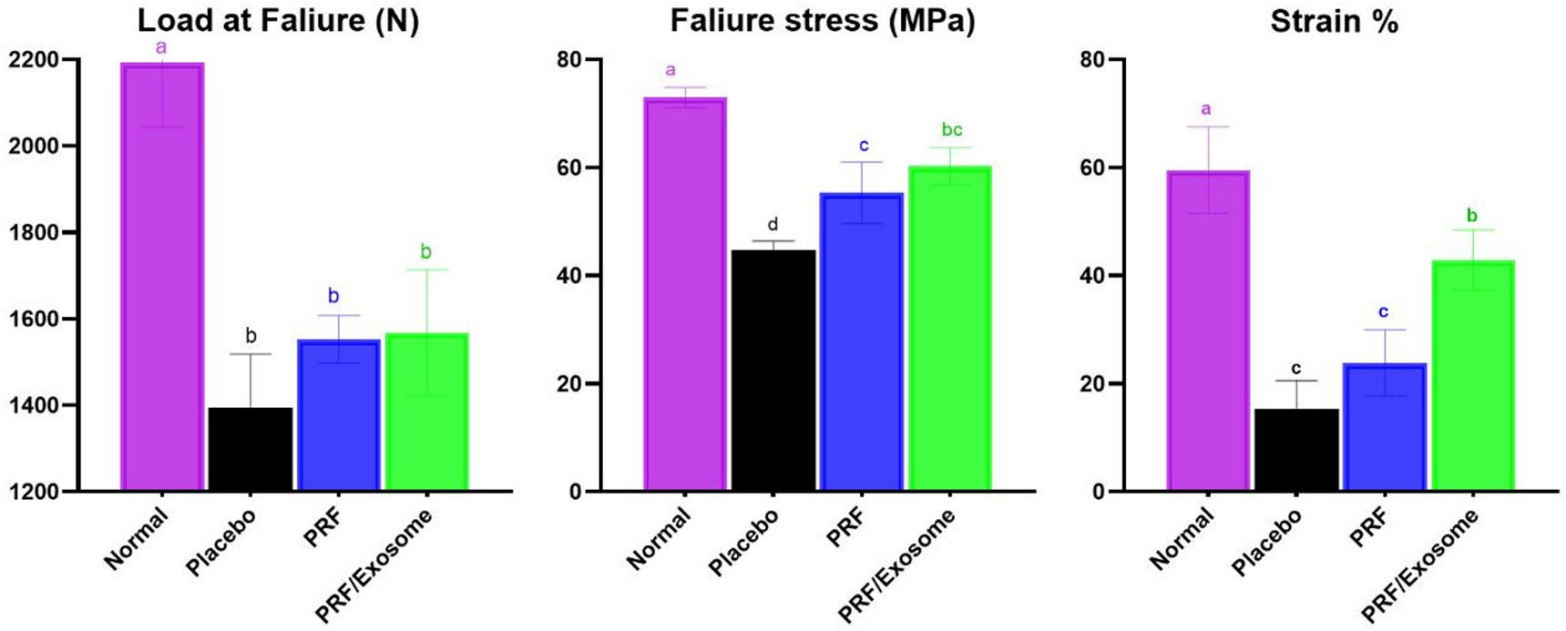
Representing the mean ± SD of failure load (N), failure stress (MPa), and strain (%) of SDFT specimens at T20 across experimental groups. Groups with different superscript letters differ significantly (*P* < 0.05).

All treated groups exhibited significantly lower failure stress values compared to the normal tendon tissue (*P* < 0.05) at T20. Among the treated groups, PRF/Exosome showed the highest failure stress values (60.24 ± 3.5 MPa), with a significant difference compared to the placebo group (44.7 ± 3.5 MPa, *P* < 0.0001) and a non-significant difference compared to the PRF group (55.3 ± 5.7 MPa, *P* = 0.098). At T20, the mean (± SD) strain % was highest in the PRF/Exosome group (42.9 ± 5.5%), significantly greater than the PRF (23.6 ± 6.1%) and placebo (15.32 ± 5.3%) groups (*P* < 0.05). When compared to the normal tendon (59.5 ± 7.9%), strain values remained significantly lower in all groups (*P* < 0.05) (Table S11, Fig. 7).

## Discussion

This study assessed the regenerative potential of I-PRF, alone or combined with MSCs-derived exosomes, in a collagenase‑induced SDF tendonitis donkey model. Donkey SDFT lesions resemble the horse condition in clinical presentation, lesion morphology, and prolonged healing^56^. Although species‑ and use‑related differences exist, most notably in tendon dimensions, biomechanical properties, and pain‑related behaviour^57,58^, the fundamental anatomical architecture and load‑sharing function of the horses and donkeys’ SDFT remain highly conserved^59^. These similarities allow careful extrapolation of the current outcomes to horses^60^. Moreover, as athletic tendinopathies in humans share similar pathogenic mechanisms, this large-animal model also provides relevant translational insight for sports medicine applications^61^.

This prospective, blinded, placebo-controlled experimental study demonstrated the superior therapeutic efficacy of intralesional I-PRF combined with MSC-derived exosomes (PRF/Exosome group) in promoting healing of collagenase-induced SDFT injuries in donkeys. Compared to the placebo group, this combination therapy significantly reduced collagen type III expression, enhanced collagen type I deposition, improved fiber echogenicity and alignment, and enhanced biomechanical performance. Moreover, the PRF/Exosome group outperformed I-PRF alone in improving the collagen I/III ratio and total histological score. I-PRF alone also conferred marked benefits compared to the placebo group, including enhancement of fiber echogenicity, a more favorable collagen I/III balance, and increased failure stress. In contrast, placebo-treated tendons exhibited disrupted architecture, hyperechoic nodularity, disorganized fibrotic matrix, and diminished mechanical strength, suggesting an inferior fibrotic healing.

In this study, clinical signs were assessed spending semi-quantitative clinical grading systems, although practical, may not provide complete accuracy. Incorporating advanced techniques, such as computerized gait analysis or thermography^62,63^, could have offered a more objective and detailed evaluation of both inflammation severity and tendon healing.

The daily clinical assessments conducted during the tendonitis induction period offer valued visions into the development of inflammation. The observed peak in pain, heat, lameness, and discomfort scores within the first few days aligns with the expected acute inflammatory response triggered by collagenase-induced tissue disruption, which leads to the rapid onset of clinical signs. This pattern is consistent with other findings, which observed similar acute inflammatory responses post-induction^64^. The significant improvement in these parameters, reaching normal or near-normal points by the end of the first week, suggests that the acute phase had largely subsided before treatment initiation. Consequently, following treatment, no significant differences in these parameters were observed among groups, as these parameters primarily reflect the transient acute inflammatory phase, which had already resolved across all groups before intervention^10^. These results are consistent with previous research on both naturally occurring and experimentally induced SDF tendonitis in donkeys and horses^60,65^. Nevertheless, some studies have reported differing outcomes, observing sustained mild elevations in these clinical parameters following treatment^66–68^.

Ultrasound remains a dependable device for noticing and spotting tendon injuries by assessing lesion size, location, and extent^69^. However, its accuracy is operator-dependent and influenced by probe pressure and measurement consistency^70^. Without strict standardization, lesion dimensions may be underestimated. Advanced imaging modalities such as MRI and CT offer greater objectivity in evaluating tendon healing^71^.

In ultrasonographic assessment of tendon healing, relying on actual lesion size has major limitations in both longitudinal follow-up within the same group and intergroup comparisons. Tendon dimensions change dynamically during inflammation and remodeling^72^, and apparent lesion enlargement may reflect overall tendon swelling rather than true pathological progression. Additionally, natural variability in tendon size across individuals^69^ can affect the clinical interpretation of lesion size; what appears mild in a large tendon may be severe in a smaller one^73^. To minimize these limitations, lesion percentage was used as a standardized parameter, allowing adjustment for individual tendon size and enabling more reliable intra-group comparisons^67^. Because baseline lesion percentages differed among groups, intergroup comparisons were based on relative changes rather than absolute values^52^. Baseline values (T0) were normalized within each group, and subsequent time points were expressed as ratios relative to T0. However, despite normalization, baseline variability in lesion percentage and T-CSA may still influence the magnitude of the observed healing response, as tendons with greater initial injury severity may exhibit different relative recovery patterns than those with milder lesions, potentially limiting direct intergroup comparisons. This should be considered when interpreting the relative treatment effects.

A persistent increase in lesion percentage and T-CSA during the repair phase indicates continued tissue damage caused by ongoing excessive inflammation. While the initial inflammatory response facilitates tissue clearance and healing by releasing growth factors and cytokines^29^, prolonged excessive inflammation disrupts collagen synthesis and shifts its production toward the weaker type III collagen instead of the stronger type I, leading to misaligned scar tissue^74^. Notably, the pattern of these increases varied significantly between groups, underscoring the differential efficacy of the treatments in regulating inflammation and ECM turnover to promote effective and controlled tendon repair.

The placebo group exhibited a significant increase in Lesion % and T-CSA up to week 8, reflecting the natural inflammatory reaction to tendon injury. In contrast, the PRF group demonstrated a slight reduction in lesion % during the first two weeks, indicating early stabilization. This effect may be related to mechanisms reported in previous studies, including PRF’s potential role in promoting macrophage polarization from the pro-inflammatory M1 to the reparative M2 phenotype and downregulation of IL-1β and IL-6^75,76^. However, due to PRF’s limited duration of cytokine release (up to 14 days)^18^, lesion % increased again at weeks 4 to 8, approaching levels similar to the placebo group. The PRF/Exosome group showed a slight, non-significant increase in lesion % up to week 4, followed by a gradual, non-significant decrease at weeks 6 and 8; this trend may reflect a more regulated healing response than both the placebo and PRF groups, as suggested by previous studies reporting potential immunomodulatory effects of MSC-derived exosomes, including modulation of macrophage polarization and downregulation of pro-inflammatory cytokines^42,77^. Importantly, these differences were mirrored by histological findings at week 8 post-treatment, where the PRF/Exosome group revealed a non-significant reduction in inflammatory markers compared to placebo and PRF groups, while both the latter retained the highest histological inflammation scores, reflecting persistent and more pronounced inflammatory activity.

The timing of lesion resolution different markedly within groups, further underlining the difference effectiveness of the treatments. In the placebo group, the delayed anechoic lesion resolution observed at week 18 indicates a prolonged inflammatory and proliferative phase typical of untreated tendon injuries. Similar findings were reported by other studies^78,79^, who noted a persistent lesion expansion up to 16 weeks in untreated tendons. The PRF/Exosome group achieved lesion resolution slightly by T18, yet demonstrated a steady decline in lesion percentage throughout the study, with a significant improvement over the PRF and placebo groups. This pattern suggests a more regulated healing response than the placebo and PRF groups^80,81^. Similarly, the PRF group exhibited lesion resolution by the end of the study, likely due to its limited ability to sufficiently regulate early inflammation compared to the combination therapy^82^.

The combined use of ultrasonographic, histological, and immunohistochemical assessments provided an integrated evaluation of tendon repair quality. In the placebo group, initial anechoic defects progressed to hyperechoic, structurally disorganized patterns consistent with fibrotic scar formation, a finding supported by palpable nodular consistency, histological evidence of collagen misalignment and a predominance of type III collagen. In contrast, the PRF/Exosome group demonstrated progressive structural improvement with lesion resolution by T18 and a favorable collagen I/III profile at T20, indicating more advanced matrix maturation despite mild residual enlargement. The PRF group exhibited intermediate findings, with persistent remodeling features and lower collagen type I expression compared to the combination therapy, suggesting incomplete structural restoration.

The changeable T-CSA pattern detected across groups reflects the dynamic nature of tendon healing, encompassing inflammation, cellular proliferation, and matrix remodeling^83^. Early increases corresponded to inflammatory edema and cellular infiltration, whereas later variations likely reflected ongoing extracellular matrix turnover and tissue reorganization. Notably, T-CSA remained above normal levels at the study endpoint in all groups, consistent with previous reports^51,52,65^. Although the placebo group approached near-normal dimensions, the PRF and PRF/Exosome groups exhibited greater residual enlargement (34% and 25%, respectively), with the PRF group demonstrating significantly higher T-CSA at T20. This persistent enlargement suggests ongoing remodeling rather than complete structural normalization.

The absence of significant differences in lameness scores among treated groups should not be interpreted as equivalent functional recovery. Routine clinical assessment during walk and trot may underestimate biomechanical deficits that become evident under higher mechanical loading, such as athletic activity^84^. Moreover, lameness alone is an insensitive indicator of tendon repair quality, as chronic tendon pain is often minimal in equine species^85^.

Although the recurrence rate of tendon injury has been suggested as a more reliable parameter for assessing long-term functionality^86^, the improved intensifying weight-bearing symmetry observed in combination-treated groups at the end of this study suggests enhanced mechanical integrity. However, more sensitive assessment tools, particularly direct tendon tensile strength measurements, remain essential for a definitive assessment of tendon repair quality^46,87^. Accordingly, both combination therapies exhibited significantly higher biomechanical performance compared to the PRF and placebo groups, with no statistical difference between them. These findings are consistent with previous studies, where MSCs-based therapies significantly improved tendon biomechanics compared to controls, although biomechanical values remained below those of native tendon tissue^51,85^.

In this study, IHC evaluation was not restricted to visual inspection; instead, quantitative assessment was performed using digital morphometric analysis with image-processing software to calculate the area percentage of positively stained regions^88^. This semi-quantitative method provides a reliable estimation of collagen distribution within defined tissue zones, although it does not reflect absolute protein concentrations^89^.

The present findings demonstrate a clear correspondence between the IHC profile and the biomechanical behavior of the repaired tendon tissue. Increased collagen type I expression in the combination-treated group at T20 paralleled its superior mechanical properties, reflecting more advanced matrix maturation and functional restoration^90^. These results are in line with previous reports showing that these therapies enhance collagen type I deposition and mechanical competence^51,85^, although variability in IHC outcomes has been described in some studies^67^.

Collagenase-induced tendonitis remains one of the most widely accepted and biologically relevant models for equine tendon injury research^46,51,60,91^. Although it does not fully replicate the progressive microdamage observed in naturally occurring cases, it reliably reproduces key pathological features, including central core lesions, matrix degradation, and a pronounced inflammatory response^51^. A recognized limitation is variability in lesion size among animals. In contrast, surgically induced models offer greater uniformity but often lack the characteristic inflammatory and degradative responses of spontaneous tendonitis^48,92^. While the influence of bacterial collagenase on the efficacy of intralesional biomaterials remains unclear, several studies have reported successful application of biologics 7–12 days after induction^51,78^.

We acknowledge that direct in-house characterization of the MSC-derived exosomes was not performed in this study. All animals received exosomes from the same production batch, ensuring batch-to-batch consistency. Biological quality and functional integrity were further supported by previously published studies using the same product^93,94^ and by the observed effects in both the current pilot and experimental study. Bone marrow-derived stem cells were selected as the source of exosomes due to their suitability for tendon regeneration^95^. Their exosomes have been reported in previous studies to enhance tenogenic gene expression and potentially modulate inflammation by promoting M2 macrophages and upregulating IL-4 and IL-10 to limit scar formation^40,96^, as well as carrying miRNAs that support extracellular matrix remodeling, thereby contributing to improved tendon structure and function^44^. Effective doses of MSC-derived exosomes in preclinical tendon models have ranged from 100 to 200 µg per lesion in rodents to approximately 500 µg per lesion in larger animals such as dogs^37,93,94^. No standardized dose has been established for equine tendons, and in this study, the exosome dose was determined based on manufacturer instructions and previous studies using the same product^94^. Nonetheless, it would have been preferable to conduct a study to evaluate the response to different doses and determine the optimal therapeutic concentration. Nonetheless, these methodological constraints should be considered when interpreting the findings.

While long-term follow-up studies are essential to evaluate re-injury rates and safe return to function, the current 20-week study was specifically designed to capture the key stages of tendon healing, including inflammation resolution, matrix remodeling, and structural maturation. This timeframe aligns with previous experimental studies and allows for a comprehensive assessment of tissue quality^51,97^.

## Conclusion

In this collagenase-induced tendonitis model in donkeys, intralesional administration of I-PRF combined with MSC-derived exosomes was associated with greater structural and functional improvement compared to the saline-treated group, as reflected by improved collagen fiber alignment, increased collagen type I deposition, decreased collagen type III expression, and enhanced tensile strength. PRF/Exosome-treated tendons also showed reduced T-CSA and better total histological scores and collagen expression compared to I-PRF therapy alone.

These findings suggest that the combined biologic approach may contribute to tendon matrix remodeling and functional repair, but the underlying mechanisms remain speculative. Further studies, particularly in naturally occurring cases, are warranted to confirm these effects and to evaluate long-term rehabilitation outcomes.

## Study limitation

The absence of long-term follow-up assessing re-injury rates and functional recovery limits the direct translational relevance of these findings to clinical practice in equine or human athletes. Future studies on naturally occurring tendonitis with long-term follow-up may help determine the durability of these findings and their implications for athletic return.

## Supplementary Information

Below is the link to the electronic supplementary material.

Supplementary Material 1

## Data Availability

All data produced or analyzed in this study are provided within this published article and its supplementary materials, including detailed results presented in the attached supplementary tables.
