# Supplementary material for: Mesenchymal stem cell derived exosomes and injectable platelet-rich fibrin enhance SDFT healing in a donkey tendonitis model
Source: Sci Rep. 2026 Apr 10;16:12013. doi: 10.1038/s41598-026-44967-7 (PMC13069002; doi:10.1038/s41598-026-44967-7)
Supplement: Supplementary file 1 — Supplementary Material 1 [file 41598_2026_44967_MOESM1_ESM.docx]

| **Table S1.** Modified ultrasound scoring system for evaluation FES & FAS **(Depuydt et al., 2021).** | | |
| --- | --- | --- |
| **Score** | **Fiber echogenicity score (FES)**  **(Transverse plane)** | **Fiber alignment score (FAS)**  **(Sagittal plane)** |
| **-1** | Hyperechoic |  |
| **0** | Normoechoic  Normal to nearly normal echogenicity | Fibers with >75 % Alignment  Normal to near normal pattern |
| **1** | Hypoechoic  25- 50% loss of normal echogenicity | Fibers with 50–74 % of normal pattern Alignment |
| **2** | Mixed echogenicity  50% anechoic and 50% normal echogenic | Fibers with 25–49% of normal pattern Alignment |
| **3** | Mostly to completely anechoic | Fibers with <25 % of normal pattern Alignment |

| **Table S2.** A modified scoring system of Movin’s grading **(Movin et al., 1997).** | | | | |
| --- | --- | --- | --- | --- |
| **Tendon repair assessment score** | | | | |
| **Score** | 0 | 1 | 2 | 3 |
| Fiber structure | Continuous, long fiber | Slightly fragmented fiber | Moderately  fragmented fiber | Severely fragmented fiber |
| Fiber arrangement | Compacted and parallel | Slightly loose and wavy | Moderately loose, wavy and cross to each other | No identifiable pattern |
| Inflammation | < 10% | 10–20% | 20–30% | > 30% |
| Angiogenesis | < 10% | 10–20% | 20–30% | > 30% |
| Cell density | Normal pattern | Slightly increase | Moderately increase | Severely increase |
| Rounding of the nuclei | Long spindle shaped | Slightly rounding | Moderately rounding | Severely rounding |
| Histopathological healing score | Grade 0: Normal | Grade I:  nearly normal | Grade II:  abnormal | Grade III:  severely abnormal |

| **Table S3.** Showing the clinical evaluation parameters during the tendonitis induction time. | | | | | | | |
| --- | --- | --- | --- | --- | --- | --- | --- |
|  | Lameness | Pain | Heat | General Appearance | Standing Posture | Resting Posture | Total Discomfort |
| Normal | 0 ^d^ | 0^e^ | 0^e^ | 0 ^c^ | 0 ^b^ | 0 ^b^ | 0 ^c^ |
| Ti1 | 5 (5-5)^a^ | 3 (3-3)^a^ | 3 (3-3)^a^ | 1 (1-2)^a^ | 1 (1-1)^a^ | 1 (1-2)^a^ | 3(3-5) ^a^ |
| Ti2 | 5 (5-5) ^a^ | 3 (3-3) ^a^ | 3 (3-3) ^a^ | 1 (1-1) ^a^ | 1 (1-1) ^a^ | 1 (1-1) ^a^ | 3(3-3) ^a^ |
| Ti3 | 4 (4-5) ^a^ | 2 (1-2) ^b^ | 2 (1-2) ^b^ | 1 (0-1) ^ab^ | 1 (0-1) ^a^ | 1 (1-1) ^a^ | 3(1-3) ^a^ |
| Ti4 | 2 (2-3) ^b^ | 1 (1-1) ^c^ | 1 (1-1) ^c^ | 1 (0-1) ^b^ | 0 (0-0) ^b^ | 0 (0-1) ^b^ | 1(0-1) ^b^ |
| Ti5 | 2 (1-2) ^bc^ | 1 (0-1) ^cd^ | 1 (0-1) ^cd^ | 0 (0-1) ^c^ | 0 (0-0) ^b^ | 0 (0-0) ^b^ | 0(0-1) ^bc^ |
| Ti6 | 1 (1-2) ^c^ | 1 (0-1) ^cd^ | 1 (0-1) ^cd^ | 0 (0-0) ^c^ | 0 (0-0) ^b^ | 0 (0-0) ^b^ | 0(0-0) ^c^ |
| Ti7=T0 | 0 (0-1) ^d^ | 0 (0-1) ^de^ | 0 (0-1) ^de^ | 0 (0-0)^c^ | 0 (0-0) ^b^ | 0 (0-0) ^b^ | 0(0-0) ^c^ |
| Test statistics | 162.305 | 157.319 | 157.319 | 122.218 | 154.507 | 161.435 | 151.946 |
| P value | 0.000 | 0.000 | 0.000 | 0.000 | 0.000 | 0.000 | 0.000 |
| Times with different superscript letters are significantly different at P<0.05, df = 7 and test statistic of Freidman test. Ti1 to Ti7 represent the tendonitis induction period (days 1 to 7 post-collagenase injection), with Ti7 corresponding to T0, the treatment initiation point. | | | | | | | |

| **Table S4**. Showing assessments of Tendon shape upon palpation and Intensifying weight bearing response under static condition before (Ti7=T0) and after treatment (T20). | | | | | |
| --- | --- | --- | --- | --- | --- |
| Tendon shape upon palpation | | | | | |
|  | Placebo | PRF | PRF/Exosome | Test statistic | P value |
| Normal | 0 (0-0)**^b^** | 0 (0-0)**^b^** | 0 (0-0)**^b^** |  |  |
| Ti7=T0 | 3 (3-3)**^a^** | 3 (3-3)**^a^** | 3 (3-3)**^a^** | 0.00 | 1.000 |
| T20 | 4 (4-4)**^a^** | 1 (0-1)**^b*^** | 0 (0-1)**^b*^** | 19.806 | 0.000 |
| Test statistic | 12.00 | 11.565 | 11.474 |  |  |
| P value | 0.002 | 0.003 | 0.003 |  |  |
| Intensifying weight bearing Response (Static examination) | | | | | |
| Normal | 0**^b^** | 0**^b^** | 0**^b^** |  |  |
| Ti7=T0 | 3 (3-3)**^a^** | 3 (3-3)**^a^** | 3 (3-3)**^a^** | 0.000 | 1.000 |
| T20 | 1 (1-1)**^ab^** | 0 (0-1)**^b^** | 0 (0-0)**^b*^** | 16.867 | 0.001 |
| Test statistic | 12.000 | 11.143 | 12.000 |  |  |
| P value | 0.002 | 0.004 | 0.002 |  |  |
| Times with different superscript letters in the same group are significantly different at p<0.05  *****there is a significant difference compared to the control group at p<0.05  **#** there is a significant difference compared to the PRF group in the same time at p<0.05 | | | | | |

| **Table S5**. Showing the ultrasonographic results of SDF tendon cross section are (T-CSA), lesion cross sectional area (L-CSA), and lesion percentage (Lesion %) before (normal) and seven days after the collagenase injection (Ti7=T0). | | | | |
| --- | --- | --- | --- | --- |
|  | T-CSA | | L-CSA | Lesion % |
|  | Normal | Ti7=T0 | Ti7=T0 | Ti7=T0 |
| Placebo | 31.2 ±0.422 | 50.2±1.3 | 21.3±1.3 | 42.2± 2.0 |
| PRF | 28.1 ±0.730 | 38.4±1.2 | 15.7±0.9 | 40.9± 2.1 |
| PRF/Exosome | 26 ±0.730 | 32.7±0.8 | 11.4±0.8 | 34.7± 3.4 |

| **Table S6**. Ultrasonographic assessment of lesion percentage (Lesion %) overtime after treatment. | | | |
| --- | --- | --- | --- |
| **Evaluation times** | **Group** | | |
|  | Placebo | PRF | PRF/Exosome |
| Ti7 = T0 | 42.2± 2.0**^efg^** | 40.9± 2.1**^bc^** | 34.7± 3.4**^ac^** |
| T1 | 53.9± 0.5**^bc^** | 38.4± 1.6**^cd*^** | 32.4± 0**^abc^** |
| T2 | 53.7± 1.8**^cd^** | 45.3± 1.6**^b*^** | 34.8± 1.5**^a*^** |
| T4 | 58.9±1.8**^a^** | 53.7± 1.2**^a^** | 35.8± 1.6**^ac*#^** |
| T6 | 58.3± 0.3**^ad^** | 52.2± 1.5**^a^** | 32.2± 2.4**^acd*#^** |
| T8 | 46.9± 1.9**^e^** | 41.3± 1.9**^bcd^** | 29.5± 2.2**^bc*^** |
| T10 | 51.5± 1.0**^c^** | 28.9± 1.6**^ef*^** | 27.5± 1.2**^bd*^** |
| T12 | 42± 0.9**^f^** | 35.2± 2.3**^de*^** | 23.1± 1.8**^b*#^** |
| T14 | 38.6± 1.3**^fg^** | 23.9± 2.1**^fg*^** | 13.3± 2.4**^e*#^** |
| T16 | 36.9± 2.2**^g^** | 20.8± 1.7**^g*^** | 8.6± 1.5**^f*#^** |
| T18 | 27.9± 1.3**^h^** | 17.9± 1.6**^g*^** | 0**^g*#^** |
| T20 | 26.9± 1.5**^h^** | 0**^h*^** | 0**^g*^** |
| Times with different superscript letters are significantly different at p<0.05.  * there is a significant difference compared to the placebo group in the same time at p < 0.05  # there is a significant difference compared to the PRF group in the same time at p < 0.05 | | | |

| **Table S7**. Showing results of tendon cross sectional area (T-CSA) across the treatment groups. | | | |
| --- | --- | --- | --- |
| **Evaluation times** | **Group** | | |
|  | Placebo | PRF | PRF/Exosome |
| Ti7=T0 | 50.2±1.3**^bc^** | 38.4±1.2**^fg^** | 32.7±0.8**^ef^** |
| T1 | 50.9 ±0.6**^b^** | 44.6± 0.9**^cd*^** | 36.5 ±0**^c*^** |
| T2 | 56.5±0.8**^a^** | 42.9± 1.0**^de^** | 33.1±0.8**^e*#^** |
| T4 | 46.4±0.8**^cd^** | 46.8±1.6**^bc*^** | 42.6±0.5**^a*^** |
| T6 | 57.1±0.9**^a^** | 44.6±0.8**^cd^** | 35.4±0.5**^d*#^** |
| T8 | 31.8±0.9**^fg^** | 39.4±0.9**^fg*^** | 38.8±0.9**^b*#^** |
| T10 | 29.9±1.5**^g^** | 53.4±0.7**^a*^** | 42.3±0.7**^a*#^** |
| T12 | 44.6±1.1**^d^** | 30.4±0.7**^h*^** | 31.7±0.6**^ef#^** |
| T14 | 49.6±0.8**^b^** | 47.6±0.8**^b*^** | 33.0±0.9**^e#^** |
| T16 | 46.2±0.7**^cd^** | 40.9±0.7**^f*^** | 31.3±0.8**^f^** |
| T18 | 34±0.9**^ef^** | 46.5±0.7**^bc*^** | 35.3±0.8**^cd*#^** |
| T20 | 35.4±0.6**^e^** | 37.6±0.8**^g*^** | 32.3±1.7**^ef*^** |
| Times with different superscript letters are significantly different at p<0.05.  ***** there is a significant difference compared to the placebo group in the same time at p < 0.05  **#** there is a significant difference compared to the PRF group in the same time at p < 0.05 | | | |

| **Table S8**. Showing results of ultrasonographic assessment of SDFT fiber echogenicity score (FES). | | | | | |
| --- | --- | --- | --- | --- | --- |
| **Evaluation Times** | **Group** | | | Kruskal-Wallis | P value |
|  | Placebo | PRF | PRF/Exosome |  |  |
| T0 | 3(3-3)**^a^** | 3(3-3)**^a^** | 3(3-3)**^a^** | 0.00 | 1.000 |
| T1 | 3(3-3)**^a^** | 3(3-3)**^a^** | 3(3-3)**^a^** | 0.00 | 1.000 |
| T2 | 3(3-3)**^a^** | 3(3-3)**^a^** | 3(3-3)**^a^** | 13.800 | 0.003 |
| T4 | 3(3-3)**^a^** | 3(2-3)**^ab^** | 3(2-3)**^ab^** | 7.667 | 0.053 |
| T6 | 2(2-3)**^ab^** | 3(2-3)**^ab^** | 3(2-3)**^ab^** | 4.343 | 0.227 |
| T8 | 2(2-3)**^ab^** | 3(2-3)**^ab^** | 2(2-3)**^abc#^** | 10.062 | 0.018 |
| T10 | 2(2-2)**^b^** | 2(2-2)**^bc^** | 2(1-2)**^bcd^** | 10.411 | 0.0.015 |
| T12 | 2(1-2)**^b^** | 2(2-2)**^bc^** | 1(1-2)**^cd*#^** | 16.611 | 0.001 |
| T14 | 2(1-2)**^b^** | 2(2-2)**^bc^** | 1(1-2)**^cd#^** | 19.806 | 0.000 |
| T16 | 1(1-2)**^b^** | 1(1-2)**^c^** | 1(0-1)**^d^** | 15.889 | 0.001 |
| T18 | -1(-1/-1)**^c^** | 1(1-1)**^c*^** | 0.5(0-1)**^d*^** | 20.240 | 0.000 |
| T20 | -1(-1/-1)**^c^** | 0(0-1)**^c*^** | 0(0-1)**^d*^** | 18.254 | 0.000 |
| Freidman test | 61.519 | 60.769 | 62.143 |  |  |
| P value | 0.000 | 0.000 | 0.000 |  |  |
| Times with different superscript letters are significantly different at p < 0.05, df = 11  ***** significant difference compared to the placebo group in the same time at p < 0.05, df = 3  **#** significant difference compared to the PRF group in the same time at p < 0.05, df = 3 | | | | | |

| **Table S9**. Showing results of ultrasonographic assessment of SDFT fiber alignment score (FAS). | | | | | |
| --- | --- | --- | --- | --- | --- |
| **Evaluation Times** | **Group** | | | Kruskal-Wallis | P value |
|  | Placebo | PRF | PRF/Exosome |  |  |
| T0 | 3(3-3)**^a^** | 3(3-3)**^a^** | 3(3-3)**^a^** | 0.000 | 1.000 |
| T1 | 3(3-3)**^a^** | 3(3-3)**^a^** | 3(3-3)**^a^** | 0.000 | 1.000 |
| T2 | 3(3-3)**^a^** | 3(3-3)**^a^** | 3(3-3)**^a^** | 0.000 | 1.000 |
| T4 | 3(3-3)**^a^** | 3(3-3)**^a^** | 3(3-3)**^a^** | 23.000 | 0.000 |
| T6 | 3(3-3)**^a^** | 3(3-3)**^a^** | 3(3-3)**^a^** | 23.000 | 0.000 |
| T8 | 3(3-3)**^a^** | 3(3-3)**^ab^** | 3(3-3)**^ab^** | 13.636 | 0.003 |
| T10 | 3(3-3)**^a^** | 3(3-3)**^ab^** | 2.5(2-3)**^ab^** | 17.250 | 0.001 |
| T12 | 3(2-3)**^ab^** | 3(2-3)**^b^** | 2(2-2)**^bc^** | 18.708 | 0.000 |
| T14 | 3(2-3)**^ab^** | 2(2-2)**^b^** | 2(2-2)**^bc^** | 19.941 | 0.000 |
| T16 | 2(2-2)**^b^** | 2(2-2)**^b^** | 2(1-2)**^bc^** | 18.553 | 0.000 |
| T18 | 2(2-2)**^b^** | 2(2-2)**^b^** | 1(1-2)**^c*#^** | 21.349 | 0.000 |
| T20 | 2(2-2)**^b^** | 1.5(1-2)**^b^** | 1(0-1)**^c^** | 19.499 | 0.000 |
| Freidman test | 56.112 | 53.907 | 59.814 |  |  |
| P value | 0.000 | 0.000 | 0.000 |  |  |
| Times with different superscript letters are significantly different at p < 0.05, df = 11  * significant difference compared to the placebo group in the same time at p < 0.05, df = 3  # significant difference compared to the PRF group in the same time at p < 0.05, df = 3 | | | | | |

| **Table S10. Quantitative analysis of collagen type I and type III area percentages in SDFT across experimental groups.** | | | | | | |
| --- | --- | --- | --- | --- | --- | --- |
| **Group** | **Evaluation times** | | | | | |
|  | **T8** | | **T14** | | **T20** | |
|  | **Collagen type III** | | | | | |
| Placebo | 14.9±0.52**^a^** | | | 6.76±0.24**^b^** | | 10.89±0.45**^c^** |
| PRF | 6.9±0.58**^a*^** | | | 12.81±0.44**^b*^** | | 1.65±0.12**^c*^** |
| PRF/Exosome | 13.2±0.52**^a*#^** | | | 3.07±0.33**^b*#^** | | 0.39±0.16**^c*#^** |
|  | **Collagen type I** | | | | | |
| Placebo |  | 0.022±0.005**^a^** | | 0.020±0.014**^a^** | | 0.494±0.122**^b^** |
| PRF |  | 0.92±0.11**^a^** | | 3.197±0.26 **^b*^** | | 10.17±0.64**^c*^** |
| PRF/Exosome |  | 0.13±0.05**^a^** | | 8.24±0.35 **^b*#^** | | 21.93±0.79 **^c*#^** |
| Times with different superscript letters are significantly different at p < 0.05  ***** significant difference compared to the placebo group in the same time at p < 0.05  **#** significant difference compared to the PRF group in the same time at p < 0.05 | | | | | | |

| **Table S11.** Comparative biomechanical properties of SDFT across experimental groups at T20. | | | |
| --- | --- | --- | --- |
|  | Load at failure (N) | Failure stress (MPa) | Strain (%) |
| Normal | 2193.04 ±149.6**^a^** | 72.9 ± 1.9**^a^** | 59.5 ±7.9**^a^** |
| Placebo | 1393.98 ±124.08**^b^** | 44.7 ± 3.5**^d^** | 15.32 ±5.3**^c^** |
| PRF | 1552.39 ±55.2**^b^** | 55.3 ± 5.7**^c^** | 23.8 ±6.1**^c^** |
| PRF/Ex | 1567.87±145.37**^b^** | 60.24 ± 3.5**^c^** | 42.9 ±5.5**^b^** |
| Groups with different superscript letters are significantly different at p < 0.05 | | | |
